# High hepatic expression of PDK4 improves survival upon multimodal treatment of colorectal liver metastases

**DOI:** 10.1038/s41416-019-0406-9

**Published:** 2019-02-27

**Authors:** Moritz J. Strowitzki, Praveen Radhakrishnan, Sandra Pavicevic, Jana Scheer, Gwendolyn Kimmer, Alina S. Ritter, Christopher Tuffs, Claudia Volz, Florian Vondran, Jonathan M. Harnoss, Johannes Klose, Thomas Schmidt, Martin Schneider

**Affiliations:** 1 Department of General, Visceral and Transplantation Surgery, University of Heidelberg, Heidelberg, Germany; 2 Regenerative Medicine and Experimental Surgery (ReMediES), Department of General, Visceral and Transplantation Surgery, Hannover Medical School, Hannover, Germany; 3 German Centre for Infection Research (DZIF), Partner Site Hannover-Braunschweig, Hannover, Germany; 4 Present Address: Conway Institute of Biomolecular and Biomedical Research, University College Dublin, Dublin, Ireland

**Keywords:** Prognostic markers, Surgical oncology, Chemotherapy, Apoptosis

## Abstract

**Background:**

Patients with borderline resectable colorectal liver metastases (CRLM) frequently receive neoadjuvant chemotherapy (NC) to reduce tumour burden, thus making surgical resection feasible. Even though NC can induce severe liver injury, most studies investigating tissue-based prognostic markers focus on tumour tissue. Here, we assessed the prognostic significance of pyruvate-dehydrogenase-kinase isoenzyme 4 (PDK4) within liver tissue of patients undergoing surgical resection due to CRLM.

**Methods:**

Transcript levels of hypoxia-adaptive genes (such as PDK isoenzymes) were assessed in the tissue of healthy liver, corresponding CRLM, healthy colon mucosa and corresponding tumour. Uni- and multivariate analyses were performed. Responses to chemotherapy upon up- or down-regulation of PDK4 were studied in vitro.

**Results:**

PDK4 expression within healthy liver tissue was associated with increased overall survival and liver function following surgical resection of CRLM. This association was enhanced in patients with NC. PDK4 expression in CRLM tissue did not correlate with overall survival. Up-regulation of PDK4 increased the resistance of hepatocytes and colon cancer cells against chemotherapy-induced toxicity, whereas knockdown of PDK4 enhanced chemotherapy-associated cell damage.

**Conclusion:**

Our findings suggest that up-regulated PDK4 expression reduces hepatic chemotherapy-induced oxidative stress and is associated with improved postoperative liver function in patients undergoing multimodal treatment and resection of CRLM.

## BACKGROUND

Many patients with colorectal cancer (CRC) develop distant organ metastases necessitating surgical resection of both the primary tumour and organ metastases.^1^ Patients with initially irresectable or borderline resectable colorectal liver metastases (CRLM) frequently receive chemotherapy to downstage the tumour burden prior to surgery, thus limiting liver resection volume and increasing success rates.^1,2^ However, chemotherapy-induced liver damage and associated effects on oncologic outcomes need to be considered.^3–6^ Chemotherapy-associated liver injury, such as hepatic steatosis, steatohepatitis and sinusoidal obstruction syndrome, occurs in up to 50% of patients receiving neoadjuvant chemotherapy (NC).^7^ Negative effects of NC on hepatic functional reserve seem to be regimen-specific.^8^ Especially platinum-based chemotherapeutics,^9,10^ which are frequently used in perioperative treatment regimens for CRLM, induce cellular oxidative stress and thereby augment DNA damage, which can lead to severe liver injury and chemotherapy-induced liver failure.^5,11–13^

Even though perioperative chemotherapy potentially harms the healthy tumour-surrounding liver tissue, the putative significance of liver-specific expression markers as prognostic indicators has been underestimated. In fact, most clinical studies investigating tissue-based molecular biomarkers focus on the primary tumour.^14–17^ There is, therefore, a strong need to develop prognostic markers that indicate the liver’s individual property to cope with aggressive combined chemotherapy and extended surgical resection of primary or secondary hepatic malignancies (such as CRLM), thus identifying patients who truly benefit from NC.

Within the given context, we could show that the loss of HIF prolyl-hydroxylase domain containing enzyme 1 (PHD1), one of the three ubiquitously expressed cellular oxygen sensors, protects murine livers from oxidative stress and liver failure in a model of acute ischaemia/reperfusion injury.^18^ Moreover, loss of PHD1 markedly reduced oxidative stress in murine peripheral muscle cells via pyruvate dehydrogenase kinase (PDK)-dependent metabolic reprogramming, thus providing protection against ischaemic stress.^19^

Here, we investigated the prognostic and predictive significance of the enzyme pyruvate dehydrogenase isoenzyme 4 (PDK4), which serves as an adaptor of hepatocyte metabolism downstream PHD1,^18,19^ in healthy liver tissue of patients with CRLM. We likewise studied putative chemoprotective effects in vitro.

## Patients and methods

The present study was conducted applying two separate patient cohorts: (i) Patients with CRLM, who underwent right hemi-hepatectomy for resection of CRLM (*n* = 62; for characteristics see Table 1; *CRLMx* cohort) and (ii) patients with CRLM, who underwent resection of the primary tumour (*n* = 33; for characteristics see Supp. Table 1; *CRCx* cohort). 36% (*n* = 12) of patients from the *CRCx* cohort received staged (*n* = 7) or simultaneous (*n* = 5) liver resection, while all other patients (*n* = 21) underwent adjuvant chemotherapy only. All patients were treated at the Department of General, Visceral and Transplant Surgery of the University Hospital Heidelberg. Clinical data from all patients were documented via a prospective database and retrospectively analysed. The study was approved by the local ethical review committee (S-649/2012) and in accordance with the Helsinki Declaration of 1975 (as revised in 1996). Detailed information regarding comorbidities, primary tumour, number of CRLMs, operations performed, chemotherapy protocols, histological signs of chemotherapy-induced liver damage (hepatic fibrosis, steatosis or steatohepatitis) and postoperative course (patient morbidity and mortality; serum levels indicating liver function and injury) was obtained from patients’ medical charts, pathological reports and surgery protocols. Clinical data from all patients were screened for perioperative hepato-toxic medication. Medication was defined as hepato-toxic applying the LiverTox database provided by the Drug-Induced Liver Injury Network (DILIN).^20^ Chemotherapy performed less than 3 months prior to surgical resection of CRLM was regarded as ‘neoadjuvant chemotherapy’ (NC). Complications were separated into intra- and postoperative complications (such as severe bleeding or bile leakage) and non-surgical complications.^3^

**Table 1:** Characteristics of all patients in the *CRLMx* cohort (*n* = 62)

		*n*	(%)
**No. of patients**		62	100.0
**Age in years** ^a^		62.0	±8.2
**Gender**	Male	40	64.5
	Female	22	35.5
**Localisation of primary tumour**	Colon	13	21.0
	Caecum	4	6.5
	Sigma	12	19.4
	Rectum	33	53.2
**UICC stadium of primary tumour**	I	4	6.5
	II	5	8.1
	III	20	32.3
	IV	31	50.0
	Unknown	2	3.2
**MSKCC score**	0	6	9.7
	I	7	11.3
	II	16	25.8
	III	29	46.8
	IV	4	6.5
	V	0	0.0
**Positive lymph node of primary tumour**	Yes	44	71.0
	No	15	24.2
	Unknown	3	4.8
**DFS interval** <**12 months**	Yes	39	62.9
	No	23	37.1
**No. of metastases**	1	23	37.1
	>1	34	54.8
	Unknown	5	8.1
**Size of metastases**	≤5 cm	38	61.3
	>5 cm	20	32.3
	Unknown	4	6.5
**Preoperative CEA-value [ng/ml]**	≤200	56	90.3
	>200	5	8.1
	Unknown	1	1.6
**Time point of metastases**	Synchronous^c^	30	48.4
	Metachronous	32	51.6
**ASA**	I	2	3.2
	II	32	51.6
	III	28	45.2
	IV	0	0.0
**Adipositas (BMI** **≥** **25)**	Yes	31	50.0
	No	30	48.4
	Unknown	1	1.6
**Diabetes**	Yes	9	14.5
	No	53	85.5
**Alcohol**	Yes	12	19.4
	No	49	79.0
	Unknown	1	1.6
**Hepato-toxic medication**	Yes	46	74.2
	No	16	25.8
**Neoadjuvant chemotherapy**	Yes	27	43.5
	No	33	53.2
	Unknown	2	3.2
**Adjuvant chemotherapy**	Yes	55	88.7
	No	7	11.3
**PDK4 mRNA expression**	Transcripts ≤ median	26	41.9
	Transcripts > median	26	41.9
	Missing values	10	16.1
**OS in months** ^b^		62.8	±40.9

*ASA* American Society of Anaesthesiologists, *DFS* disease-free survival, *MSKCC* Memorial Sloan-Kettering Cancer Center, *OS* overall survival, *PDK* pyruvate dehydrogenase kinase, *UICC* Union for International Cancer Control

^a^Data are given as median ± standard deviation

^b^Data are given as mean ± standard deviation

^c^Diagnosed before or within 3 months after resection of the primary tumour

### Histology and immunohistochemistry

Paraffin-embedded tissues were sectioned at 5 µm thickness, dewaxed and rehydrated in xylene and graded ethanol series. For immunohistochemistry, antigens were retrieved with Target Retrieval Solution (Dako, Hamburg, Germany), blocked with serum from the same species the secondary antibody was raised in (Vector Laboratories, Burlingame, USA), and incubated overnight with the following primary antibodies: PDK4 (1:400; ab89295; Abcam, Cambridge, UK) or 8-hydroxy-2′-deoxyguanosine (8-OHdG; 1:200; NB600-1508; Novus Biologicals, Littleton, USA). The following day, appropriate secondary antibody was added and amplified with TSA^TM^ Indirect (Perkin Elmer, Rodgau, Germany), before DAB-labelling (Dako). Quantification of positively stained areas was carried out by two independent, blinded investigators, using an Axiostar Plus Microscope (Carl Zeiss, Jena, Germany) and ImageJ software (National Institutes of Health, Bethesda, USA).

### Quantitative real-time PCR

Total RNA was isolated using RNeasy Mini Kit (Qiagen, Hilden, Germany) and transcribed into cDNA with Improm-II-Reverse Transcription System (Promega, Mannheim, Germany). qPCR was performed using Light Cycler ®480 SYBR Green Master (Roche, Mannheim, Germany) and specific primers (Supp. Table 2). ß-Actin served as a housekeeping gene for cell culture experiments and 18S was used with human tissue samples. Obtained *cp*-values which were defined as statistical outliers were removed from the data set. Finally, the patient collectives were stratified according to the obtained expression patterns (≤ or > median) of the different hypoxia adaptive genes (*PDK*, pyruvate dehydrogenase kinase; *PFKL*, 6-phosphofructokinase, liver type; *PPAR-α*, peroxisome proliferator-activated receptor alpha; *UCP-2*, mitochondrial uncoupling protein).

### Western blotting

Whole-cell lysates were prepared in RIPA lysis buffer (Merck Millipore, Burlington, USA). Primary antibodies against PDK4 (human: 1:200; Abcam; ab38242; mice: 1:500; Novus Biologicals; NBP1-07047), vinculin (1:2000; Abcam; ab129002) and tubulin (Cell Signaling Technology; Denver; USA; 2148) were used. After development with an HRP-conjugated secondary antibody, semiquantitative analysis was performed applying ImageJ software (NIH). The quotient of areas under the curve (AUC) for PDK4 and housekeeping protein (AUC PDK4/AUC tubulin or vinculin) is representative of the amount of PDK4 protein present in the loaded sample. In humans, results from several western blots were pooled to the corresponding group (*n* = 8 patients in each group; lowest PDK4 transcripts versus highest PDK4 transcripts). In murine hepatocytes treated with increasing concentrations of fenofibrate, data from three independent experiments were pooled.

### Cell culture experiments

All cell lines were purchased from ATCC (LGC Standards, Wesel, Germany). Human hepatocytes (HepG2 cells) were kindly provided by Dr. Anna-Lena Scherr and Dr. Bruno Koehler (NCT, University of Heidelberg, Germany). Murine hepatocytes (AML12 cells) were cultured in equal ratio of DMEM/F12 (Sigma-Aldrich, Taufkirchen, Germany) and DMEM high glucose 25 mM (Sigma-Aldrich) medium with insulin, transferrin and selenium (ITS-G, Thermo Fisher Scientific, Waltham, USA), supplemented with 10% FCS and 1% penicillin/streptomycin. Human HepG2 and HCT 116 cells were maintained in DMEM/F12 with 10% FCS and 1% penicillin/streptomycin. Murine CT 26 cells were cultured in RPMI-1640 medium (Sigma-Aldrich) with 10% FCS and 1% penicillin/streptomycin. Cells were grown at 37 °C in a humidified 5% CO_2_ incubator.

Fenofibrate (Sigma-Aldrich) was used to induce PDK4 expression.^21^ Cells were treated with increasing concentrations of fenofibrate (50 and 200 µM). 5-fluorouracil (500 µM; 5-FU) or oxaliplatin (100 µM; Ox) were used as chemotherapeutic agents and cells were treated for 24 and 48 h to determine time-dependent effects on cellular characteristics. Cells treated with dimethyl sulfoxide (DMSO; Sigma-Aldrich) were used as corresponding controls. All cell culture experiments were repeated at least three times. Representative graphs from one representative out of three consistent experiments were depicted.

### Transient knock-in and knock-down of PDK4

Cells were plated at a density of 2 × 10^6^ cells on 10 cm dishes. Transient PDK4 knock-in was performed using a CRISPR activation plasmids (human: sc-401355-ACT; mouse: sc-424220-ACT; Santa Cruz, Heidelberg, Germany), mixed with UltraCruz® Transfection Reagent (Santa Cruz), according to the manufacturer’s protocol. A control CRISPR activation plasmid was used as a negative control. Transfected cells were incubated for 48 h under normal culture conditions, and subsequently used for experimental assays.

Transient PDK4 knock-down was performed using a pool of siRNA sequences (FlexiTube GeneSolution; Qiagen) targeting murine PDK4 mRNA (catalogue number GS27273; SI01374156, SI01374149, SI01374142, SI01374135) or human PDK4 mRNA (catalogue number GS5166; SI02660056, SI02660049, SI02629067, SI00040467), together with Lipofectamine RNAiMAX (Thermo Fisher Scientific). AllStars Negative Control siRNA (Qiagen; SI03650318) served as a control. Cells were collected 48 h after transfection and treated with 500 µM 5-FU or 100 µM Ox for 24 and 48 h. Transfection efficiency for both knock-in and knock-down was determined via RT-qPCR (Supp. Figure 1A–D).

### Determination of cell viability (WST1 and DAPI staining), proliferation (BrdU) and apoptosis (TUNEL)

Cell viability was determined by water soluble tetrazolium (WST)-1 assay (Sigma-Aldrich). 5000 Cells were seeded in 96-well plates, allowed to attach and treated as described above. WST-1 reagent diluted 1:10 in growth medium was added to each well. After incubation at 37 °C in a 5% CO_2_ incubator for 3 h, absorbance at 450 nm was measured using a microplate reader (Tecan, Crailsheim, Germany). As a non-metabolic readout for cell viability we stained AML12 cells with the fluorescence nucleus dye DAPI (Invitrogen; ProLong™ Gold Antifade Mountant with DAPI) and counted cells with and without signs of cytotoxicity (viable cells in percent).^22^ Cell proliferation was measured using a colorimetric cell proliferation ELISA (Roche), according to the manufacturer’s protocol. This assay measures 5-bromo-2′-deoxyuridine (BrdU) incorporated in replicating cells. The reaction product was quantified by measuring the absorbance at 450 nm using a microplate reader. Double strand breaks as indicator of cell apoptosis were measured via TUNEL assay (R&D Systems, Minneapolis, USA) in AML12 cells treated with 5-FU. The reaction product was quantified by measuring the absorbance at 450 nm.

### Assessment of reactive oxygen species (ROS)

Generation of intracellular ROS was determined by applying the ROS-Glo H_2_O_2_ Assay (Promega), which measures the level of produced hydrogen peroxide (H_2_O_2_) in cells. H_2_O_2_ is a common end-product of oxidative metabolism with a long half-life. For the assay, transfected cells were plated at a density of 1 × 10^4^ cells per well in 96-well plates and treated with 500 µm 5-FU for 48 h. H_2_O_2_ substrate solution was added 6 h prior to the end of treatment time. Luminescence signal was measured with a microplate reader.

### Fluorescence microscopy (MitoTracker Red CM-H2Xros)

For fluorescence microscopy, transfected AML12 cells were grown on coverslips and treated with 500 µM 5-FU for 48 h. Prior to imaging 250 nM MitoTracker Red^™^ (CM-H2Xros, Thermo Fisher Scientific) was added to the cells and incubated for 45 min. Afterwards cells were washed and fixed with 4% formaldehyde (Thermo Fisher Scientific) for 15 min. Cells were counterstained with DAPI (Thermo Fisher Scientific). Microscopic pictures were taken with an Axiostar Plus Microscope (Carl Zeiss).

### Statistics

Statistical analysis was carried out with SPSS 1 version 18.2 (SPSS®, Chicago, USA) and GraphPad Prism version 7 (GraphPad® Software, La Jolla, USA). Statistical analysis of two groups was performed using Student’s *t*-test. *χ*^2^ testing or ANOVA, with adequate post-hoc analysis, were applied to assess differences in frequencies of histological signs for chemotherapy-associated liver injury or continuous data sets of more than two groups, respectively. The Kaplan–Meier method was applied to estimate overall survival rates of all patients, and survival data were compared by log-rank test. For the validation of prognostic factors regarding overall survival and multivariate analysis, we performed stepwise Cox proportional hazard regression as previously described.^23^ Prognostic factors for multivariate analysis were chosen by their *P*-values (<0.1) obtained during univariate analysis. Patients with missing values were automatically excluded from multivariate analysis.^23^ Univariate analysis regarding hypoxia adaptive genes was restricted to four genes showing the highest expression in liver tissue (Supp. Figure 2A and B). We performed two-sided testing and considered a *P*-value of <0.05 as significant.

## Results

### Patient characteristics

In the main patient cohort (*n* = 62; *CRLMx*, comprising patients undergoing right hemi-hepatectomy for CRLM), the median age was 62.0 years and two-thirds of the patients were men. The majority of patients were originally diagnosed with advanced-stage rectal cancer and had a Memorial Sloan-Kettering Cancer Center (MSKCC) Score of III (for detailed information see Table 1). Half of the patients were diagnosed with multiple metastases, affecting more than one segment of the liver. While 44% of the patients received NC (*n* = 27), a majority of 89% underwent adjuvant chemotherapy. At the time of surgery healthy liver tissue of patients with NC showed no significant increase of histological signs, such as liver fibrosis, steatosis or steatohepatitis which can be associated with chemotherapy-induced liver damage (Supp. Table 3). Mean overall survival was 62.8 months from the date of primary diagnosis (for detailed information see Table 1).

In a second patient cohort (*n* = 33; *CRCx*, comprising patients with CRLM undergoing resection of the primary tumour), the median age was 66.4 years and two-thirds of the patients were men. All patients from this cohort were originally diagnosed with advanced-stage colorectal cancer (UICC IV) and suffered from synchronous liver metastases. None of the patients received NC or preoperative radiation of the primary tumour. However, 75% of patients underwent postoperative chemotherapy. Mean overall survival was 32.4 months from the date of primary diagnosis (for detailed information see Supp. Table 1).

### PDK4 expression in CRLM and in primary colorectal tumours

**Fig. 1:**
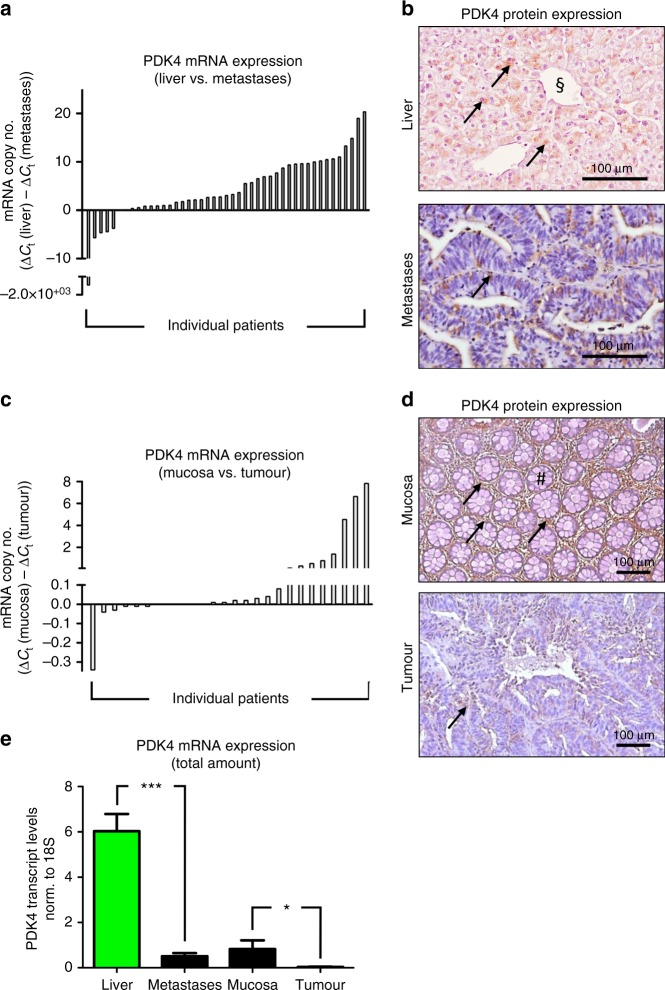
PDK4 expression in CRLM and in primary colorectal tumours. **a** Real-time PCR analysis revealing higher expression of PDK4 mRNA in healthy liver tissue compared to colorectal liver metastases. Each bar represents an individual patient; bar values [ΔCt (liver) − ΔCt (metastases)] indicate the difference of PDK4 mRNA expression between healthy liver tissue and corresponding metastases (*n* = 45 patients; *CRLMx* cohort). **b** Representative immunostainings revealing PDK4 protein expression (arrows) in healthy liver tissue (top) and liver metastases (bottom). The sectional symbol indicates central vein. **c** Real-time PCR revealing higher expression of PDK4 mRNA in healthy colon mucosa compared to primary colorectal tumours. Each bar represents an individual patient; bar values [ΔCt (mucosa) − ΔCt (tumour)] represent the difference of PDK4 mRNA between healthy mucosa and corresponding tumour (*n* = 26 patients; *CRCx* cohort). **d** Representative immunostainings revealing PDK4 protein expression (arrows) in healthy colon mucosa (top; hashtag depicting crypt) and primary colorectal tumours (bottom). **e** Real-time PCR analysis revealing PDK4 transcript levels in liver tissue, liver metastases, healthy colon mucosa and primary colorectal tumours. *P < 0.05; ***P < 0.001; *n* = 53 (liver) versus 51 (metastases)/28 (mucosa) versus 31 (tumour).

Expression analysis of PDK4 in patients undergoing surgical resection of CRLM (*CRLMx* cohort) revealed that PDK4 mRNA expression was consistently higher in tumour-surrounding liver tissue than in tissue derived from corresponding CRLM (Fig. 1a, e). Consistently, immunohistochemistry revealed more abundant expression of PDK4 protein in healthy liver tissue than in corresponding CRLM tissue (Fig. 1b; Supp. Figure 3A). Spatial expression of PDK4 protein within liver tissue was mostly restricted to hepatocytes (Supp. Figure 3A). Western blotting confirmed that PDK4 protein levels were likewise reduced in patients displaying low hepatic PDK4 mRNA transcript levels (Supp. Figure 3B).

For comparison, we likewise assessed the expression of PDK4 within primary cancer sites. These analyses were performed in patients suffering CRLM, who underwent surgical resection of the primary tumour (*CRCx* cohort). In a majority of cases, PDK4 transcript expression was likewise significantly higher in healthy colon mucosa than in corresponding primary colorectal tumour tissue (Fig. 1c, e). Immunohistochemistry confirmed more abundant expression of PDK4 protein in healthy mucosa tissue than in primary tumour tissue (Fig. 1d).

Taken together, in patients suffering advanced CRC, PDK4 expression was significantly more abundant in healthy liver and mucosa tissue than in corresponding CRLM- or primary tumour tissue.

### High hepatic, but not tumoural PDK4 expression is associated with improved outcomes upon neoadjuvant chemotherapy and surgical resection of CRLM

**Fig. 2:**
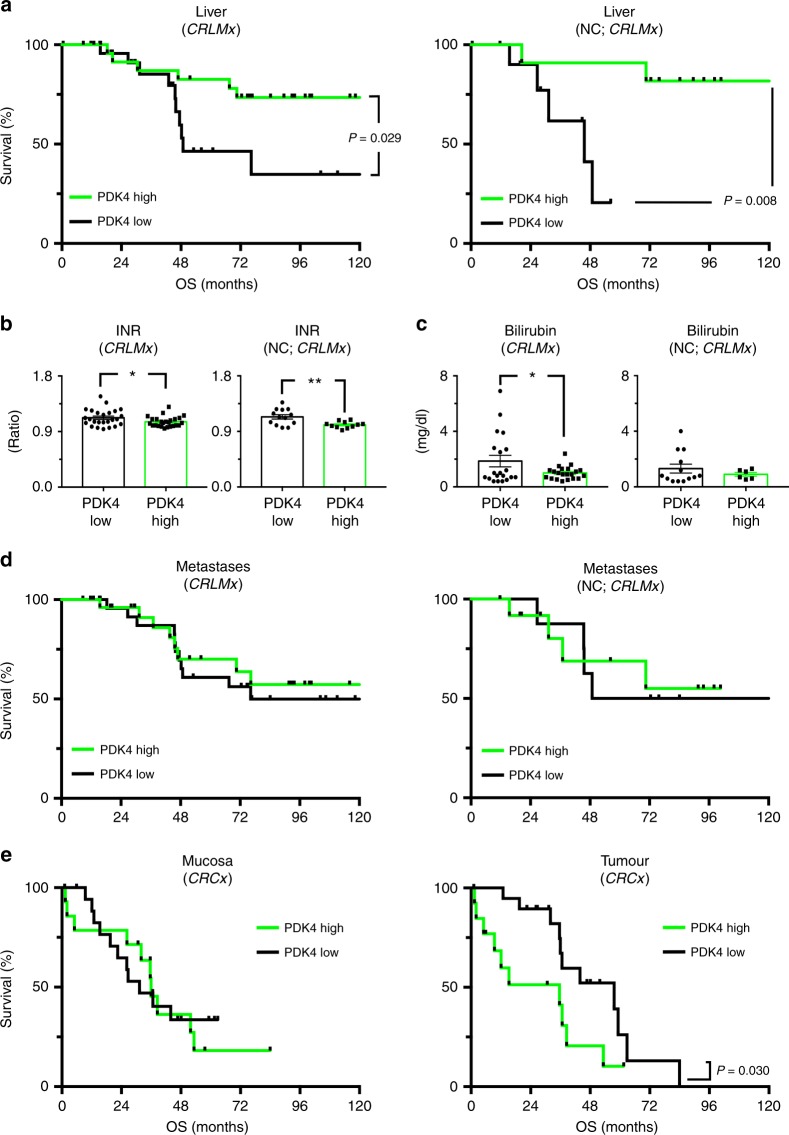
High hepatic, but not tumoural expression of PDK4 is associated with improved survival in patients undergoing surgical resection of CRLM. **a** Left: Kaplan–Meier curves revealing the survival of patients with high (green curves) versus low (black curves) intrahepatic expression of PDK4 (*n* = 52; all patients from CRLMx cohort). Right: Kaplan–Meier curves indicating the survival of patients with high versus low intrahepatic PDK4 expression, treated with neoadjuvant chemotherapy (NC) plus right hemi-hepatectomy (*n* = 23 patients from *CRLMx* cohort). Note increased survival of patients with high intrahepatic PDK4 expression. **b**, **c** Serum markers indicating liver function in all patients from the *CRLMx* cohort (left panels in **b**, **c**; *n* = 52) and in the subgroup with NC plus resection (right panels in **b**, **c**; *n* = 23 patients from *CRLMx* cohort), assessed 1 week after right hemi-hepatectomy. Note lower INRs and bilirubin concentrations indicating improved liver function, in patients with high hepatic PDK4 expression (green bars) compared to those with low PDK4 mRNA expression (black bars). *P < 0.05; **P < 0.01. **d** Left: Kaplan–Meier curves revealing the survival of patients with high versus low intrametastatic expression of PDK4 (*n* = 52; all patients from *CRLMx* cohort). Right: Kaplan–Meier curves indicating the survival of patients with high versus low intrametastatic PDK4 expression, undergoing NC plus right hemi-hepatectomy (*n* = 21 patients from *CRLMx* cohort). **e** Kaplan–Meier curves revealing the survival of patients with high versus low PDK4 expression within healthy colon mucosa (left) and within primary colorectal tumours (right; *n* = 33 patients from *CRCx* cohort). Note decreased survival of patients with high intratumoural PDK4 expression (right).

In order to assess its putative clinical significance, PDK4 expression in the diverse tumour and normal tissue compartments was correlated with oncologic outcome data. Interestingly, high PDK4 transcript expression in healthy liver tissue was associated with increased patient survival (Fig. 2a, left). This effect was already significant when looking at all patients within the *CRLMx* cohort, and particularly striking in those undergoing NC followed by hemi-hepatectomy (Fig. 2a, right). Indeed, in those patients, univariate analysis revealed hepatic PDK4 expression, the number of liver metastases and the occurrence of intraoperative complications as prognostic factors associated with overall survival (Table 2). Strikingly, in multivariate analysis, hepatic PDK4 transcript expression proved to be an independent prognostic factor, indicating disease-specific survival in CRLM patients treated with NC prior to resection (Table 3; Fig. 2a, right). In CRLM patients undergoing resection without NC, however, hepatic PDK4 expression levels did not significantly influence overall survival (Supp. Figure 4A). In the entire patient cohort (all patients), intraoperative complications and the size of liver metastases proved to be independent prognostic factors (Table 3).

**Table 2:** Univariate analysis of prognostic factors associated with overall survival of all patients (*n* = 62) and of patients with NC (*n* = 27) in the *CRLMx* cohort

		All patients (*n* = 62)			Patients with NC (*n* = 27)		
		*P*-value	OS in months		*P*-value	OS in months	
			Mean	95% CI		Mean	95% CI
**Age in years**		0.917			0.429		
	≤ Median		139.4	104.9–173.8		83.1	51.1–115.1
	> Median		97.1	77.1–117.0		92.5	67.1–118.0
**Gender**		0.837			0.884		
	Male		96.3	79.2–113.3		93.3	68.6–118.0
	Female		118.1	75.7–160.5		66.0	38.3–93.7
**Localisation of primary tumour**		0.247			0.858		
	Colon		65.3	46.3–84.3		75.6	47.4–103.9
	Caecum		51.7	39.4–63.7		45.6	45.6–45.6
	Sigma		96.8	73.5–120.1		60.8	36.9–84.7
	Rectum		123.4	85.4–161.5		91.8	63.0–120.6
**MSKCC score**							
	Stratified	0.487			0.631		
	<III		122.8	83.7–161.9		62.1	39.8–84.3
	≥III		92.7	72.8-112.6		95.9	70.5–121.3
	Positive lymph node	0.066			0.129		
	Yes		80.2	66.2–94.2		na	na
	No		145.8	95.8–195.8		na	na
	DFS interval <12 months	0.095			0.349		
	Yes		88.3	69.4–107.2		97.3	73.8–120.7
	No		134.1	92.4–175.8		47.2	73.3–120.7
	No. of metastases	0.002			0.115		
	1		151.9	106.3–197.5		93.1	80.3–105.9
	>1		80.2	61.5–98.9		83.2	56.1–110.3
	Size of metastases	0.087			0.314		
	≤5 cm		90.9	72.2–109.6		80.4	54.9–106.0
	>5 cm		163.6	125.4–201.7		112.7	79.9–145.5
	Preoperative CEA-value [ng/ml]	0.748			0.282		
	≤200		117.6	83.1–152.2		95.2	72.5–117.9
	>200		86.5	64.2–108.8		45.5	45.5–45.5
**Liver metastases**							
	Time of appearance	0.635			0.843		
	Synchronous^a^		87.4	67.8–107.0		87.7	61.5–114.0
	Metachronous		119.4	83.7–155.1		88.4	57.1–119.6
	Hepatic relapse	0.418			0.803		
	Yes		77.8	58.2–97.3		68.5	46.4–90.6
	No		98.5	77.5–119.5		94.8	62.3–127.7
**ASA**		0.645			0.460		
**Adipositas (BMI** **≥** **25)**		0.133			0.272		
	Yes		107.5	89.8–125.1		105.4	73.9–137.0
	No		115.5	77.2–153.8		75.8	50.0–101.6
**Diabetes**		0.838			0.486		
	Yes		87.6	66.5–108.7		48.8	48.8–48.8
	No		117.1	82.1–152.1		95.0	72.3–117.8
**Alcohol**		0.457			0.116		
	Yes		107.8	76.5–139.1		122.3	90.1–154.5
	No		112.0	79.0–145.0		61.7	45.7–77.8
**Hepato-toxic medication**		0.692			0.446		
	Yes		100.3	84.2–116.3		97.0	72.5–121.6
	No		123.7	72.0–175.4		63.3	34.7–91.8
**Neoadjuvant chemotherapy**		0.493			na		
	Yes		92.7	70.5–114.8		na	na
	No		122.9	84.9–160.9		na	na
**Hepatic fibrosis**		0.663			0.471		
	Yes		104.0	80.6–127.5		70.8	48.2–93.4
	No		130.8	99.6–161.9		88.5	64.1–113.0
**Hepatic steatosis**		0.325			0.314		
	Yes		123.3	86.3–160.4		84.2	57.3–111.1
	No		79.8	58.2–101.4		81.6	59.3–103.9
**Steatohepatitis in grades**		0.280			0.341		
	0		na	na		na	na
	I		na	na		na	na
	II		na	na		na	na
	III		na	na		na	na
**Time on ICU in days**		0.295			0.169		
	≤Median		125.9	88.0–163.8		105.9	80.6–131.2
	>Median		76.1	57.0–95.4		56.9	73.3–118.0
**Complications**							
	Intraoperative complications	0.048			0.057		
	Yes		51.8	34.0–69.5		41.9	31.3–52.4
	No		123.6	87.6–159.6		101.5	78.2–124.9
	Postoperative complications	0.136			0.412		
	Yes		87.5	68.7–106.3		84.1	55.1–113.1
	No		134.0	90.4–175.5		79.4	59.8–99.0
**Comorbidities**							
	Cardiovascular risk factors	0.381			0.047		
	Yes		87.3	71.4–103.1		na	na
	No		114.1	80.4–147.8		na	na
	COPD	0.520			0.459		
**Hypoxia adaptive genes**							
	PDK4	0.029			0.008		
	Transcripts ≤ median		74.0	54.0–94.0		40.8	31.6–50.0
	Transcripts > median		169.5	137.8–201.2		123.4	100.7–146.0
	PFKL	0.070			0.092		
	Transcripts ≤ median		75.9	57.2–94.7		42.6	32.7–52.4
	Transcripts > median		163.5	128.6–198.4		97.8	74.6–121.0
	PPAR-α	0.799			0.128		
	Transcripts ≤ median		86.9	63.4–105.3		56.5	36.0–77.1
	Transcripts > median		140.0	104.6–175.3		107.3	80.5–134.1
	UCP-2	0.978			0.609		
	Transcripts ≤ median		134.1	94.8–173.4		64.6	46.2–83.0
	Transcripts > median		97.2	77.5–116.9		99.5	71.0–128.0

*ASA* American Society of Anaesthesiologists, *BMI* body mass index, *CI* confidence interval, *COPD* chronic obstructive pulmonary disease, *DFS* disease-free survival, *MSKCC* Memorial Sloan-Kettering Cancer Center, *na* not available (all cases are censored), *NC* neoadjuvant chemotherapy, *OS* overall survival, *PDK* pyruvate dehydrogenase kinase, *PFKL* 6-phosphofructokinase, liver type, *PPAR-α* peroxisome proliferator-activated receptor alpha, *UCP-2* mitochondrial uncoupling protein, *UICC* Union for International Cancer Control

^a^Diagnosed before or within 3 months after resection of the primary tumour

**Table 3:** Multivariate analysis of prognostic factors associated with overall survival of all patients (*n* = 37) and of patients with NC (*n* = 20) in the *CRLMx* cohort

	*P*-value	RR	95% CI
**All patients (*n*** = 37)^a^			
**MSKCC score**			
Positive lymph node	0.282	3.584	0.3–43.9
Yes			
No			
DFS interval <12 months	0.997	0.695	0.2–2.9
Yes			
No			
No. of metastases	0.573	1.325	0.3–5.5
1			
>1			
Size of metastases	0.004^b^	0.120	0.0–0.7
≤5 cm			
>5 cm			
**Complications**			
Intraoperative complications	0.019^b^	7.769	1.7–36.6
Yes			
No			
**Hypoxia adaptive genes**			
PDK4	0.197	0.283	0.0–2.0
Transcripts ≤ median			
Transcripts > median			
PFKL	0.576	1.900	0.3–14.0
Transcripts ≤ median			
Transcripts ≤ median			
**Patients with NC (*n*** = 20)^a^			
**Complications**			
Intraoperative complications	0.821	1.271	0.2–9.2
Yes			
No			
**Comorbidities**			
Cardiovascular risk factors	0.970	na	na
Yes			
No			
**Hypoxia adaptive genes**			
PDK4	0.037	0.097	0.0–0.9
Transcripts ≤ median			
Transcripts > median			
PFKL	0.750	0.118	0.1–21.3
Transcripts ≤ median			
Transcripts ≤ median			

*CI* confidence interval, *DFS* disease-free survival, *MSKCC* Memorial Sloan-Kettering Cancer Center, *na* not available (all cases are censored), *NC* neoadjuvant chemotherapy, *PDK* pyruvate dehydrogenase kinase, *PFKL* 6-phosphofructokinase, liver type, *RR* relative risk

^a^Cases with missing values not included

^b^Confirmation by Cox backward regression analysis

To further investigate the putative significance of PDK4 as a predictive biomarker, indicating the impact of NC-associated adverse effects (such as drug-induced liver injury) on overall survival, we additionally stratified the *CRLMx* cohort into patients with low versus high intrahepatic PDK4 expression. Interestingly, NC was associated with decreased overall survival only in patients with low hepatic PDK4 expression, but not in those with high hepatic PDK4 expression (Supp. Figure 4B).

On the basis of these data, we speculated that hepatic PDK4 expression provides survival advantages by protecting against cumulative functional liver damage, inflicted by NC plus major hepatectomy in CRLM patients. To further characterise such putative hepato-protective effects, we analysed serum markers indicating postoperative liver function and -injury 1 week after hemi-hepatectomy. Indeed, high hepatic expression of PDK4 was associated with reduced international normalised ratios (INR; prothrombin time), indicating enhanced liver synthetic function (Fig. 2b).^24^ This effect was observed in all patients from the *CRLMx* cohort, but pronounced in those having undergone preoperative NC (Fig. 2b, right). Furthermore, serum bilirubin concentrations were reduced in patients with high hepatic PDK4 expression, albeit this difference was only significant when looking at all patients within the *CRLMx* cohort (Fig. 2c). Serum markers of liver cell damage (such as glutamate-oxaloacetate transaminase [GOT] or glutamate-pyruvate transaminase [GPT]) were not significantly affected by hepatic PDK4 expression (Supp. Figure 4C). Moreover, high hepatic PDK4 expression had only marginal effects regarding histological signs of chemotherapy-associated liver damage (Supp. Table 3). We also tested whether diabetes or treatment with NC, which may by itself affect hepatic PDK4 expression,^25,26^ induced PDK4 expression in our patient cohort. Notably, hepatic PDK4 transcript expression was comparable in patients with or without diabetes (Supp. Figure 5A) and did likewise not differ between patients with or without NC (Supp. Figure 5B). Overall, these findings support the notion that high hepatic expression of PDK4 confers enhanced liver function, without significantly affecting liver cell damage 1 week after major hepatectomy in CRLM patients.

Having established a link between hepatic PDK4 expression and survival upon NC and resection of CRLM, we sought to investigate putative effects of intratumoural PDK4 expression on patient prognosis. PDK4 mRNA expression levels within liver metastases (CRLM tissue) affected overall survival neither in all patients from the *CRLMx* cohort (Fig. 2d, left), nor in the patient subgroup having undergone NC (Fig. 2d, right). Further analyses were carried out in patients from the *CRCx* cohort (comprising patients with CRLM undergoing resection of the primary tumour). Interestingly, high PDK4 transcript expression within primary colorectal tumours was rather associated with impaired survival rates (Fig. 2e, right). PDK4 transcript expression within healthy mucosa (surrounding primary tumours) did not affect patient survival (Fig. 2e, left).

Taken together, we found that hepatic, but not tumoural expression of PDK4 was significantly associated with improved survival in patients undergoing NC and major hepatectomy for treatment of CRLM. This effect was accompanied with clinically improved postoperative liver function after extended liver resection.

### PDK4 expression confers chemoprotection to hepatocytes

We speculated that enhanced survival of CRLM patients with high hepatic PDK4 expression was linked to chemoprotective effects, conferred by the metabolic regulator PDK4. Indeed, initial in vitro experiments revealed that exposure to 5-fluorouracil (5-FU) or oxaliplatin (Ox), two chemotherapeutics that are commonly combined for treatment of CRLM, significantly increased PDK4 transcript levels in murine hepatocytes (AML12 cells) (Supp. Figure 6A and B).

**Fig. 3:**
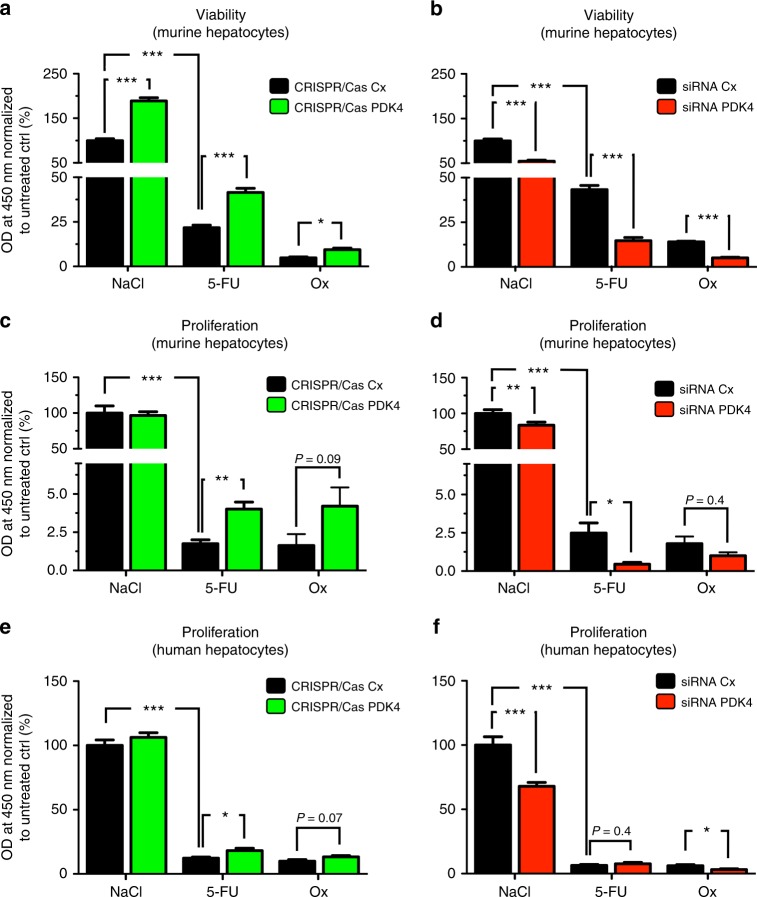
PDK4 confers chemoprotection to hepatocytes. **a**, **b** WST-1 assays revealing the viability of murine hepatocytes (AML12) exposed to 5-fluorouracil (5-FU; 500 µM), oxaliplatin (Ox, 100 µM) or vehicle control (NaCl). Note that 5-FU- or Ox-induced impairment of cell viability is significantly attenuated by up-regulation of PDK4 gene function (CRISPR/Cas PDK4) compared to control (CRISPR/Cas Cx) (**a**) but aggravated by siRNA-mediated knockdown of PDK4 (siRNA PDK4) compared to control (siRNA Cx) (**b**). **c**, **d** BrdU assays revealing the proliferation of AML12 cells exposed to 5-FU, Ox or NaCl. Note that 5-FU- or Ox-induced impairment of proliferation is significantly attenuated by up-regulation of PDK4 gene function (CRISPR/Cas PDK4) compared to control (CRISPR/Cas Cx) (**c**) but aggravated by siRNA-mediated knockdown of PDK4 (siRNA PDK4) compared to control (siRNA Cx) (**d**). **e**, **f** BrdU assays revealing the proliferation of human hepatocytes (HepG2) exposed to 5-FU, Ox or NaCl. Note that 5-FU- or Ox-induced impairment of proliferation is significantly attenuated by up-regulation of PDK4 gene function (CRISPR/Cas PDK4) compared to control (CRISPR/Cas Cx) (**e**) but aggravated by siRNA-mediated knockdown of PDK4 (siRNA PDK4) compared to control (siRNA Cx) (**f**). Each graph in (**a**–**f**) represents pooled data from three independent experiments. *P < 0.05; **P < 0.01; ***P < 0.001; *n* = 3.

To further assess the effects of altered PDK4 gene expression on 5-FU- and Ox-induced hepatocyte-toxicity, we applied CRISPR/Cas 9 gene editing or siRNA-mediated silencing to up- or down-regulate PDK4 expression, respectively, in murine and human hepatocytes. Exposure to 5-FU or Ox expectedly reduced the viability of murine hepatocytes (AML12 cells; Fig. 3a, b). Remarkably, induction of PDK4 gene function significantly increased cell viability upon treatment with 5-FU or Ox (Fig. 3a), whereas inhibition of PDK4 gene function further aggravated 5-FU- or Ox-induced impairment of hepatocyte viability (Fig. 3b). As an alternative, non-metabolic readout of cell viability, we applied DAPI staining, and subsequently counted cells with and without signs of cytotoxicity.^22^ Consistent with the results outlined above, chemotherapy-induced impaired cell viability was increased upon CRISPR/Cas-mediated up-regulation of PDK4 expression (Supp. Figure 6C).

Anti-proliferative effects of 5-FU or Ox on murine hepatocytes were consistently attenuated upon induction of PDK4 expression (Fig. 3c), and interference with PDK4 expression further aggravated cytostatic effects of 5-FU and Ox in these cells (Fig. 3d). Furthermore, 5-FU-induced apoptosis of murine hepatocytes was significantly attenuated upon CRISPR/Cas-mediated up-regulation of PDK4 expression (Supp. Figure 7A), whereas interference with PDK4 expression enhanced 5-FU-induced apoptosis in these cells (albeit this latter effect did not quite reach statistical significance; Supp. Figure 7B). Similar analyses were carried out applying human hepatocytes (HepG2 cells), and revealed comparable results (Fig. 3e, f; Supp. Figure 7C).

Collectively, these in vitro data support the notion that hepatocellular PDK4 expression is protective against liver cell damage inflicted by prolonged chemotherapy.

### Pharmacological induction of PDK4 attenuates chemotherapy-induced cytotoxicity

PDK4 acts as a metabolic regulator downstream peroxisome proliferator-activated receptor alpha (PPAR-α).^19^ Indeed, within our patient cohort (*CRLMx* cohort, see above), we observed a significant linear correlation between hepatic mRNA expression levels of PPAR-α and PDK4 (Supp. Figure 7D). Given this link, gene and protein expression of PDK4 (among other PPAR-α targets) can be pharmacologically induced by the PPAR-α agonist fenofibrate (Supp. Figure 8A and B).^21^ We thus assessed whether fenofibrate treatment, putatively via up-regulation of PDK4, can likewise abrogate chemotherapy-induced cytotoxity in hepatocytes and primary tumour cells.

**Fig. 4:**
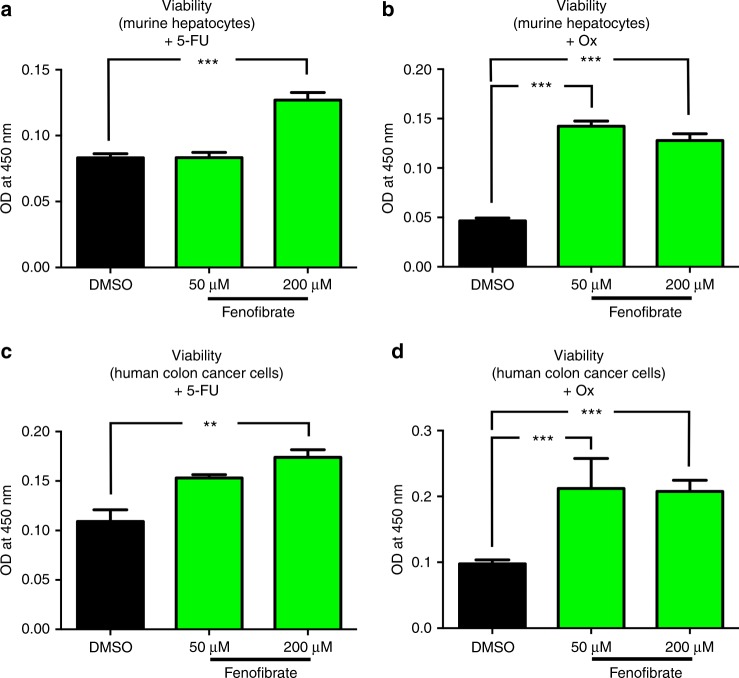
Pharmacological induction of PDK4 attenuates chemotherapy-induced cytotoxicity. **a**, **b** WST-1 assays revealing that the viability of murine hepatocytes (AML12 cells) exposed to 5-FU (500 µM) (**a**) or Ox (100 µM) (**b**) is significantly enhanced by simultaneous treatment with fenofibrate (50 or 200 µM). **c**, **d** WST-1 assays revealing that the viability of human colon cancer cells (HCT 116) exposed to 5-FU (500 µM) (**c**) or Ox (100 µM) (**d**) is significantly enhanced by simultaneous treatment with fenofibrate (50 or 200 µM). Each graph in (**a**–**d**) represents pooled data from three independent experiments. **P < 0.01; ***P < 0.001; *n* = 3.

Fenofibrate dose-dependently increased the viability of murine hepatocytes (AML12 cells) upon exposure to 5-FU or Ox (Fig. 4a, b), and likewise significantly increased hepatocyte proliferation under treatment with Ox (Supp. Figure 8C). Remarkably, these effects were not restricted to liver cells, since fenofibrate exerted chemoprotective effects in murine (CT 26 cells) and human colon cancer cell lines (HCT 116 cells). Indeed, fenofibrate treatment increased the viability of these cancer cells upon exposure to 5-FU or Ox (Fig. 4c, d; Supp. Figure 8D). This finding is consistent with our observation that high PDK4 mRNA expression within primary tumours was associated with decreased survival in human patients (Fig. 2e, right).

### Up-regulation of PDK4 attenuates chemotherapy-induced oxidative stress

**Fig. 5:**
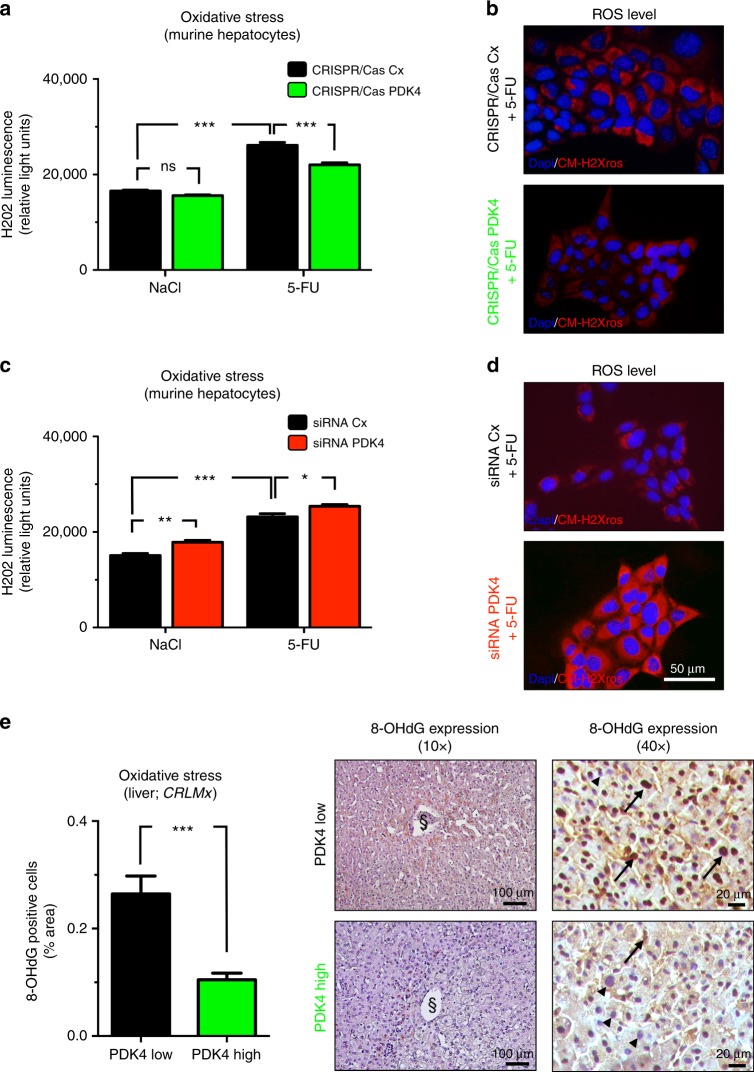
Up-regulation of PDK4 attenuated chemotherapy-induced oxidative stress. **a**–**d** Hydrogen peroxide (H_2_O_2_) luminescence assays (**a**, **c**) and representative fluorescence labelling for CM-H2Xros (red) and DAPI (cell nuclei, blue) (**b**, **d**) to indicate oxidative stress in murine hepatocytes (AML12) exposed to 5-FU (500 µM) or vehicle control (NaCl). Note that 5-FU-induced oxidative stress is significantly attenuated by up-regulation of PDK4 gene function (CRISPR/Cas PDK4) compared to control (CRISPR/Cas Cx) (**a**, **b**), but aggravated by siRNA-mediated knockdown of PDK4 (siRNA PDK4) compared to control (siRNA Cx) (**c**, **d**). Graphs (**a**, **c**) represent pooled data from three independent experiments. *P < 0.05; **P < 0.01; ***P < 0.001; *n* = 3. **e** Representative 8-OHdG immunostainings (right) and histomorphometric quantification (left) of liver tissue from CRLM patients (from *CRLMx* cohort), indicating significantly attenuated oxidative stress in patients with high hepatic PDK4 expression (green bar and bottom panels) compared to those displaying low hepatic PDK4 expression (black bar and upper panels). Note reduced nuclear abundance (arrowheads in right panels) of 8-OHdG adducts (arrows in right panels) in patients with high hepatic PDK4 expression. *n* = 8 per group; ***P < 0.001. The sectional symbol indicates central vein.

It has previously been demonstrated that the induction of PDKs can attenuate hepatocellular oxidative stress by reducing cellular glucose oxidation and oxygen consumption.^18,19^ To assess the putative relevance of this mechanism in PDK4-dependent modulation of chemotherapy-induced cytotoxicity, we applied H_2_O_2_ luminescence and CM-H2Xros fluorescence microscopy to screen for oxidative stress in 5-FU treated hepatocytes (AML12 cells) upon up- or downregulation of PDK4 expression in vitro. 5-FU-exposure expectedly increased oxidative stress levels in hepatocytes (Fig. 5a, c). Strikingly, CRISPR/Cas-mediated up-regulation of PDK4 expression resulted in significantly attenuated cellular oxidative stress upon 5-FU exposure (Fig. 5a, b). By contrast, siRNA-mediated interference with PDK4 gene function slightly, but significantly enhanced chemotherapy-induced oxidative stress (Fig. 5c, d).

In order to assess the putative relevance of these mechanisms in liver tissue from CRLM patients, we performed immunostainings for 8-hydroxy-2′-deoxyguanosine (8-OHdG), a tissue marker of deoxyguanosine-oxidation, indicating oxidative stress. Indeed, liver tissue from patients with low hepatic PDK4 expression (*CRLMx* cohort) stained more strongly for 8-OHdG than liver tissue from those with high PDK4 expression, indicating attenuated hepatocellular oxidative stress upon elevated hepatic PDK4 expression (Fig. 5e, left panel). Indeed, patients with low hepatic PDK4 expression showed more intense nuclear 8-OHdG staining compared to those with high PDK4 expression (Fig. 5e, right panels).

Collectively, these findings suggest that PDK4 induces chemoprotection in hepatocytes via attenuated oxidative stress.

## Discussion

In the present study, we demonstrate that high transcript levels of PDK4 within healthy liver tissue of patients with CRLM are associated with increased overall survival and postoperative liver function. Moreover, up-regulation of PDK4 gene function as well as pharmacological induction of PDK4 attenuated chemotherapy-induced cell damage as indicated by enhanced cell viability and decreased apoptosis. By contrast, down-regulation of PDK4 expression was accompanied with increased chemotherapy-induced cell damage in vitro. These effects were most probably conferred by reduced oxidative cell stress due to up-regulation of PDK4.

So far, most trials investigating tissue-based prognostic markers in patients with advanced CRC focussed on tumour tissue, obtained from the primary cancer site or from metastatic lesions.^14,15,17^ However, postoperative liver dysfunction, due to chemotherapy-induced oxidative stress and cellular apoptosis, may significantly limit the overall survival of patients undergoing liver resection for treatment of CRLM.^5,27^ In our opinion, the putative significance of liver-specific prognostic and predictive markers, which are detectable in healthy tissue obtained from CRLM patients, has been underestimated. Specifically, we hypothesise that hepatic expression of key metabolic markers, such as PDK4, indicates the liver’s property to cope with aggressive combined chemotherapy and extended surgical resection, thus predicting prognosis in patients undergoing multimodal therapy of CRLM. Indeed, we could show that, firstly, PDK4 mRNA expression was significantly higher in healthy liver and colon mucosa compared to tissue derived from CRLM or primary colon tumours; that, secondly, increased hepatic expression of PDK4 represented an independent prognostic factor concerning overall survival of patients receiving NC; and, thirdly, that PDK4 expression was likewise associated with improved postoperative liver function in patients undergoing surgical resection of CRLM. Remarkably, hepatic PDK4 expression appeared to affect survival prognosis most strikingly in patients who had received NC. Moreover, NC was only associated with decreased overall survival in patients with a low hepatic PDK4 expression but did not affect overall survival in patients with high hepatic PDK4 expression, altogether suggesting that PDK4 confers hepatoprotective effects upon multimodal and surgical treatment of CRLM. However, the putative significance of PDK4 as a suitable biomarker to identify CRLM patients that benefit best from perioperative chemotherapy has to be interpreted with caution, since NC treatment on its own was not significantly associated with impaired overall survival in our patient cohort. Therefore, the clinical applicability of hepatic PDK4 expression as a predictive biomarker for chemotherapy-induced liver damage might be limited.

Rather consistent with our findings regarding PDK4 expression in liver tissue and CRLM, a recent study revealed that PDK4 expression was significantly more abundant in tumour-adjacent liver tissue than in primary liver tumours.^28^ In contrast to our findings, however, a previous translational study reported significantly up-regulated PDK4 mRNA expression within CRC tissue compared to healthy colon mucosa.^29^ This may in part be due to differences in patient characteristics between the two studies. Indeed, the patient cohort analysed in our present study (*CRCx* cohort) comprised solely patients suffering metastasised CRC, while the above-mentioned study excluded patients with chemotherapy or radiotherapy in their clinical history. Remarkably, our findings concerning PDK4 transcript expression were backed up with immunohistochemical analyses, confirming that PDK4 transcript levels correlated with PDK4 protein expression in our patient samples.

PDK isoenzymes exert a central role concerning the metabolic switch that is present in tumour cells, commonly referred to as Warburg effect.^30,31^ On the one hand, inhibition of PDK4 can reportedly reduce proliferation and increase apoptosis in lung cancer cells,^32^ recapitulating our findings obtained in human and murine hepatocytes. Consistently, pharmacological or siRNA-mediated interference with PDK4 function reportedly inhibited proliferation of bladder cancer cells.^33^ More importantly, a recent study demonstrated that genetic loss of PDK4 resulted in spontaneous cell death of human hepatocellular carcinoma cells (HCC), which was accompanied by mitochondrial dysfunction and increased production of ROS.^34^ Moreover, PDK4-deficient mice suffered from increased chemically-induced liver injury, which could be prevented by inhibition of the NF-kB subunit p65.^34^ These recent findings highlight the potential of PDK4 over-expression to alleviate chemically-induced liver injury and to reduce hepatic ROS levels, which is confirmed by the results outlined in our present study.^34^ On the other hand, however, overexpression of PDK4 has been demonstrated to attenuate adenosine triphosphate (ATP) levels and suppress proliferation in breast cancer cells.^35^ This would contrast with our findings, which revealed that pharmacological induction of PDK4 induces viability and proliferation in both hepatocytes and colon cancer cells. Importantly, a recent study revealed that baseline hepatocyte proliferation was induced in PDK4-deficient mouse livers, most probably via increased cell cycle progression.^36^ However, while important, these results were obtained applying genetically modified mice, and may thus be only indirectly applicable to findings from our study, which rely on observations in human patients and in vitro settings. These in part conflicting results regarding the effect of ATP levels on tumour growth and proliferation may also be explained by the fact that extracellular ATP levels can act both growth stimulating and growth inhibiting, depending on receptors expressed by the tumour cells themselves and on the immune cell-mediated anti-tumoural host response.^37^

PDK4, which is a downstream target of the metabolic master regulator PPAR-α,^38^ is up-regulated upon loss of HIF prolyl-hydroxylase domain containing enzyme 1 (PHD1), a cellular oxygen sensing enzyme.^19^ It has previously been demonstrated that up-regulation of PDK4, which occurred in a PHD1- and PPAR-α-dependent fashion, critically reduced oxidative stress in skeletal muscle, thus protecting mice from ischaemic muscle necrosis.^19^ Accordingly, we found that chemotherapy-induced oxidative stress in hepatocytes was attenuated upon genetic up-regulation of PDK4. Likewise, we detected attenuated oxidative stress levels in liver tissue from patients with high hepatic PDK4 expression. Notably, oxidative stress-induced ROS-signalling is known to induce hepatocyte death and liver injury, which can be prevented by superoxide scavengers.^39^ A recent clinical study demonstrated that patients with CRLM displayed increased levels of oxidative DNA damage within liver metastases as compared to primary tumour tissue, and that oxidative cell stress, as detected by 8-OHdG immunohistochemistry, was increased upon chemotherapy in these patients.^40^ Notably, however, this study did not address the effects of chemotherapy on oxidative stress levels within healthy liver or colon tissue from CRLM patients.^40^ Collectively, these findings support our hypothesis that PDK4 represents a key modulator of oxidative stress-induced hepatocyte damage, which occurs upon chemotherapeutic treatment.

Consistent with our findings, PDK4 expression can reportedly be induced by hepatotoxic agents such as chemotherapeutic drugs, and attenuate liver injury inflicted by such substances.^26^ However, conflicting evidence likewise exists. A recent preclinical study revealed that interference with PDK4 gene function attenuated cisplatin-induced acute kidney injury in mice.^41^ Further analysis revealed reduced oxidative stress levels in primary kidney cells isolated from PDK4-deficient kidneys.^41^ This contrasts with our findings, which revealed chemoprotective effects conveyed by up-regulation of PDK4. Most likely, these apparent differences regarding the role of PDK4 in the protection of cell viability and proliferation are due to cell-type specific properties. Indeed, hepatocytes and tumour cells are highly metabolically active. Tumour cells, in particular, rely on aerobic glycolysis, which can be facilitated by PDK enzymes.^42^ For this reason, PDK4 may represent a promising therapeutic target in cancer medicine.^31^ Down-regulation of PDK4, for instance, increases apoptosis in human colon cancer cells.^43^ In fact, and in sharp contrast with its significance in liver tissue, we found that tumoural PDK4 expression was rather associated with impaired prognosis of patients suffering metastasised CRC.

In conclusion, our findings indicate that high hepatic expression of PDK4 is associated with increased survival of patients undergoing liver resection due to CRLM, likely by improving liver function that can be affected by surgical reduction of functional liver volume on the one hand, and chemotherapy-induced hepatocyte damage on the other. The predictive significance of hepatic PDK4 expression, which may prove helpful in selecting patient subgroups benefitting most from aggressive multimodal therapy of CRLM, however, has to be interpreted with caution.

## Supplementary information

Supplementary material - clean

Supp. Figure 1

Supp. Figure 2

Supp. Figure 3

Supp. Figure 4

Supp. Figure 5

Supp. Figure 6

Supp. Figure 7

Supp. Figure 8

## Data Availability

The datasets generated and/or analysed during the current study are not publicly available but are available from the corresponding author on reasonable request.
