# Supplementary material for: High hepatic expression of PDK4 improves survival upon multimodal treatment of colorectal liver metastases
Source: Br J Cancer. 2019 Feb 27;120(7):675–88. doi: 10.1038/s41416-019-0406-9 (PMC6461828; doi:10.1038/s41416-019-0406-9)
Supplement: Supplementary file 1 — Supplementary material - clean [file 41416_2019_406_MOESM1_ESM.docx]

High hepatic expression of PDK4

improves survival upon multimodal treatment

of colorectal liver metastases

Moritz J. Strowitzki^1,2^, Praveen Radhakrishnan^1^, Sandra Pavicevic^1^, Jana Scheer^1^, Gwendolyn Kimmer^1^, Alina S. Ritter^1^, Christopher Tuffs^1^, Claudia Volz^1^, Florian Vondran^3,4^, Jonathan M. Harnoss^1^, Johannes Klose^1^, Thomas Schmidt^1^

and Martin Schneider^1^

1. Department of General, Visceral and Transplantation Surgery, University of Heidelberg, Heidelberg, Germany.
2. Current address: Conway Institute of Biomolecular and Biomedical Research, University College Dublin, Dublin, Ireland.
3. Regenerative Medicine and Experimental Surgery (ReMediES), Department of General, Visceral and Transplantation Surgery, Hannover Medical School, Hannover, Germany.
4. German Centre for Infection Research (DZIF), partner site Hannover-Braunschweig, Hannover, Germany.

These authors contributed equally to this work

**SUPPLEMENTARY FIGURE LEGENDS**

**Supp. Figure 1. *(A, C)*** real-time PCR analysis revealing significantly increased PDK4 mRNA expression upon CRISPR/Cas9 gene editing (CRISPR/Cas PDK4) compared to control (CRISPR/Cas Cx) in murine (AML12; *A*) and human hepatocytes (HepG2; *C*). ***(B, D)*** real-time PCR analysis revealing significantly decreased PDK4 mRNA expression upon siRNA mediated silencing (siRNA PDK4) compared to control (siRNA Cx) in murine (AML12; *B*) and human hepatocytes (HepG2; *D*). Graphs in *A-D* represent pooled data from three independent experiments. **P*<0.05; ***P*<0.01; ****P*<0.001; *n*=3.

**Supp. Figure 2. *(A, B)*** real-time PCR analysis revealing transcript expression patterns of hypoxia-adaptive and PHD1-modulated genes in human liver tissue (pooled, *A*) and of individual patients (3D-plot, *B*) (*n*=62 patients from *CRLMx* cohort; *EPO*, erythropoietin; *GCK*, glucokinase; *PDK*, pyruvate dehydrogenase kinase; *PFKL*, 6-phosphofructokinase, liver type; *PGC-1α*, Peroxisome proliferator-activated receptor gamma coactivator 1 alpha; *PHD*, hypoxia-inducible factor (HIF) prolyl-hydroxylase; *PPAR-*$\alpha$, peroxisome proliferator-activated receptor alpha; *UCP-2*, Mitochondrial uncoupling protein).

**Supp. Figure 3. *(A)*** Representative image of PDK4 immunohistochemistry from human liver (patient from *CRLMx* cohort), revealing high PDK4 protein expression in healthy liver tissue (#) compared to low PDK4 expression in CRLM (§). Dotted line depicts the border between healthy liver tissue and metastasis. ***(B)*** Western Blotting (one representative blot, left) and densitometric analysis (right) revealing significantly increased PDK4 protein expression in patients with high hepatic PDK4 mRNA levels compared to patients with low PDK4 mRNA levels (**P*<0.05; *n*=8 per group).

**Supp. Figure 4. *(A)*** Kaplan–Meier curve indicating the survival of patients with high (green curve) versus low (black curve) intrahepatic PDK4 expression, treated with right hemihepatectomy in absence of neoadjuvant chemotherapy ($\emptyset$ NC) (*n*=27 patients from *CRLMx* cohort). ***(B)*** Left: Kaplan–Meier curves revealing the survival of patients with low intrahepatic PDK4 expression (PDK4 low) treated with (NC, red curves) or without neoadjuvant chemotherapy ($\emptyset$ NC, black curves) (n=26; patients from *CRLMx* cohort). Right: Kaplan–Meier curves revealing the survival of patients with high intrahepatic PDK4 expression (PDK4 high) treated with (red curves) or without ($\emptyset$, black curves) neoadjuvant chemotherapy (NC) (n=24; patients from *CRLMx* cohort). Note decreased survival only of patients with low intrahepatic PDK4 expression associated with NC treatment. ***(C)*** Serum levels of albumin, gamma glutamyl transpeptidase ($\text{γ}$GT), glutamate-oxaloacetate transaminase (GOT) and glutamate-pyruvate transaminase (GPT) in all patients from the *CRLMx* cohort (left panels; *n*=52) and in the subgroup with NC plus resection (right panels; *n*=24 patients from *CRLMx* cohort), assessed one week after right hemihepatectomy.

**Supp. Figure 5. *(A, B)*** real-time PCR analysis revealing comparable PDK4 transcript levels in liver tissue from patients with or without ($\emptyset$) diabetes mellitus (*A*), and in those with or without ($\emptyset$) neoadjuvant chemotherapy (NC; *B*). *n*=6 (diabetes) vs. 46 ($\emptyset$ diabetes) / 22 (NC) vs. 27 ($\emptyset$ NC).

**Supp. Figure 6. *(A, B)*** real-time PCR analysis revealing a dose-dependent increase of PDK4 transcript expression in murine hepatocytes (AML12 cells) upon treatment with 5-fluorouracil (5-FU; *A*) or oxaliplatin (Ox; *B*). ***(C)*** Cell nucleus staining with DAPI revealing the viability of murine hepatocytes (AML12 cells) exposed to 5-fluorouracil (5-FU; 500µM), oxaliplatin (Ox, 100µM) or vehicle control (NaCl). Note that Ox-induced impairment of cell viability is significantly attenuated by up-regulation of PDK4 gene function (CRISPR/Cas PDK4) compared to control (CRISPR/Cas Cx). Graphs represent pooled data from three independent experiments. **P*<0.05; ***P*<0.01; ****P<0.0001; *n*=3.

**Supp. Figure 7. *(A-C)*** TUNEL assays revealing apoptosis of murine (AML12 cells; *A,B*) and human hepatocytes (HepG2; *C*) exposed to 5-fluorouracil (5-FU; 500µM). Note attenuated apoptosis upon up-regulation of PDK4 gene function (CRISPR/Cas PDK4) compared to control (CRISPR/Cas Cx) (*A*), but enhanced apoptosis upon siRNA-mediated knockdown of PDK4 (siRNA PDK4) compared to control (siRNA Cx) (*B*)*.* Graphs represent pooled data from three independent experiments. **P*<0.05; *n*=3. ***(D)*** Correlation analysis revealing a significant linear correlation between intrahepatic expressions of PPAR-$\alpha$ and PDK4 mRNA in patients from the *CRLMx* cohort.

**Supp. Figure 8. *(A)*** real-time PCR analysis revealing a significant increase of PDK4 transcript expression in human hepatocytes (HepG2 cells) upon treatment with 200µM fenofibrate. Graph represents pooled data from five independent experiments. **P<0.01; n=5. ***(B)*** Western Blotting (one representative blot, left) and densitometric analysis (right) revealing significantly increased PDK4 protein expression in murine hepatocytes (AML12 cells) after treatment with 200µM fenofibrate. ***(C)*** BrdU assay revealing that the proliferation of murine hepatocytes (AML12 cells) exposed to Ox (100µM) is significantly enhanced by simultaneous treatment with fenofibrate (50µM or 200µM). ***(D)*** WST-1 assay revealing that the viability of murine colon cancer cells (CT26) exposed to 5-FU (500µM) is significantly enhanced by simultaneous treatment with fenofibrate (50µM or 200µM). Graphs in *B-D* represent pooled data from three independent experiments. **P*<0.05; ***P*<0.01; ****P*<0.001; *n*=3.

**SUPPLEMENTARY TABLES**

**Supp. Table 1** Characteristics of all patients in the *CRCx* cohort (n=33).

|  |  | n | (%) |
| --- | --- | --- | --- |
| **No. of patients** |  | 33 | 100.00 |
| **Age in years^§^** |  | 66.39 | ±10.50 |
| **Gender** | male | 23 | 69.70 |
|  | female | 10 | 30.30 |
| **Localization of primary tumour** | Colon | 13 | 39.39 |
|  | Caecum | 4 | 12.12 |
|  | Sigma | 7 | 21.21 |
|  | Rectum | 6 | 18.18 |
| **UICC stadium of primary tumour** | I | 0 | 0.00 |
|  | II | 0 | 0.00 |
|  | III | 0 | 0.00 |
|  | IV | 33 | 100.00 |
| **MSKCC Score** | 0 | 0 | 0.00 |
|  | I | 1 | 3.03 |
|  | II | 5 | 15.15 |
|  | III | 7 | 21.21 |
|  | IV | 6 | 18.18 |
|  | V | 4 | 12.12 |
|  | unknown | 10 | 30.30 |
| **preoperative CEA-value (ng/ml)** | ≤ 200 | 18 | 54.55 |
|  | > 200 | 11 | 33.33 |
|  | unknown | 4 | 12.12 |
| **Time point of metastases** | synchronous^#^ | 33 | 100.00 |
|  | metachronous | 0 | 0.00 |
| **ASA** | I | 1 | 3.03 |
|  | II | 14 | 42.42 |
|  | III | 7 | 21.21 |
|  | IV | 0 | 0.00 |
|  | unknown | 11 | 33.33 |
| **Neoadjuvant chemotherapy** | yes | 0 | 0.00 |
|  | no | 33 | 100.00 |
| **Adjuvant chemotherapy** | yes | 25 | 75.76 |
|  | no | 0 | 0.00 |
|  | unknown | 8 | 24.24 |
| **PDK4 mRNA expression** | transcripts ≤ median | 20 | 60.6 |
|  | transcripts > median | 13 | 39.4 |
| **OS in months*** |  | 32.36 | ±20.30 |

§Data are given as median ± standard deviation; *Data are given as mean ± standard deviation; ^#^Diagnosed before or within 3 months after resection of the primary tumour; *ASA* American Society of Anaesthesiologists; *MSKCC* Memorial Sloan-Kettering Cancer Center; *OS* overall survival; *PDK* pyruvate dehydrogenase kinase; *UICC* Union for International Cancer Control.

**Supp. Table 2** mRNA primer sequences for q-RT-PCR.

| **Gene** | **Synonym** | **Forward Sequence** | **Reverse Sequence** |
| --- | --- | --- | --- |
| *Actb** | ß-actin | 5’GCTGTATTCCCCTCCATCGT3’ | 5’AGGTGTGGTGCCAGATCTTC3’ |
| *ACTB* | ß-actin | 5’ATGTGGCCGAGGACTTTGATT3’ | 5’AGTGGGGTGGCTTTTAGGATG3’ |
| *EGLN2* | PHD1 | 5’AGATTGCCTGGGTGGAAG3’ | 5’TGCCTGGGTAACACGCC3’ |
| *EGLN1* | PDH2 | 5’CGACCTGATACGCCACTGTA3’ | 5’CCTTGGCATCCCAGTCTTTA3’ |
| *EGLN3* | PDH3 | 5’TCAGATCGTAGGAACCCACAC3’ | 5’GAACAAGGCCAGCAGATT TC3’ |
| *EPO* | EPO | 5’CTATGCCTGGAAGAGGATGG3’ | 5’TGGGAAGAGTTGACCAACAG3’ |
| *GCK* | GCK | 5’TGGAGGAGATGCAGAATGTG3’ | 5’CCCATGTACTTGCCACCTATG3’ |
| *PDK1* | PDK1 | 5’CGCTGGGTAATGAGGATTTG3’ | 5’TTACCACCCCCAGCCAGAGG3’ |
| *Pdk4** | PDK4 | 5'TCGACCCAAACTGTGATGTG3' | 5'TCGAAGAGCATGTGGTGAAG3' |
| *PDK4* | PDK4 | 5’GCCTTTGAGTGTTCAAGGATG3’ | 5’GAAGGCTGATTTTCCTGGTG3’ |
| *PFKL* | PFKL | 5’ACACCCGTGTAACTGTGCTG3’ | 5’AGCCGCACTGACTGGTCC3’ |
| *PPARA* | PPAR-$\alpha$ | 5’TCACAAGTGCCTTTCTGTCG3’ | 5’TAGGCCTCGTAGATTCTCTGGG3’ |
| *PPARGC1A* | PGC1-$\alpha$ | 5’TCTGACCACAAACGATGACC3’ | 5’TCAAATGGGGAACCCTTG3’ |
| *UCP2* | UCP2 | 5’AAAGCACCGTCAATGCCTAC3’ | 5’TGATGAGGTCATAGGTCACCAG3’ |
| *RNA18SN2* | 18S | 5'AAACGGCTACCACATCCAAG3' | 5'CCTCCAATGGATCCTCGTTA3' |

***mouse genes*; EPO* erythropoietin; *GCK* glucokinase; *PDK* pyruvate dehydrogenase kinase; *PFKL* 6-phosphofructokinase, liver type; *PGC-1*$\alpha$ Peroxisome proliferator-activated receptor gamma coactivator 1 alpha; *PHD* hypoxia-inducible factor (HIF) prolyl-hydroxylase; *PPAR-*$\alpha$ peroxisome proliferator-activated receptor alpha; *S* Svedberg units; *UCP-2* Mitochondrial uncoupling protein.

**Supp. Table 3** Effects of neoadjuvant chemotherapy and intrahepatic PDK4 expression on histological signs of chemotherapy-associated liver injury.

|  |  | **All patients (n=60)** | | | | |  |  | **All patients (n=52)** | | | | |  |
| --- | --- | --- | --- | --- | --- | --- | --- | --- | --- | --- | --- | --- | --- | --- |
|  |  | Patients without NC | |  | Patients with NC | |  |  | Low PDK4 | |  | High PDK4 | |  |
|  |  | n | (%) |  | n | (%) | **P-value** |  | n | (%) |  | n | (%) | **P-value** |
| **Hepatic fibrosis** |  |  |  |  |  |  | 0.155 |  |  |  |  |  |  | 1.000 |
|  | yes | 13 | 39.4 |  | 6 | 22.2 |  |  | 8 | 30.8 |  | 8 | 30.8 |  |
|  | no | 20 | 60.6 |  | 21 | 77.8 |  |  | 18 | 69.2 |  | 18 | 69.2 |  |
| **Hepatic steatosis** |  |  |  |  |  |  | 1.000 |  |  |  |  |  |  | 0.244 |
|  | yes | 22 | 66.7 |  | 18 | 66.7 |  |  | 19 | 73.1 |  | 15 | 57.7 |  |
|  | no | 11 | 33.3 |  | 9 | 33.3 |  |  | 7 | 26.9 |  | 11 | 42.3 |  |
| **Steatohepatitis** |  |  |  |  |  |  | 0.596 |  |  |  |  |  |  | 0.226 |
|  | 0 | 19 | 57.6 |  | 15 | 55.6 |  |  | 16 | 61.5 |  | 14 | 53.8 |  |
|  | I | 10 | 30.3 |  | 10 | 37.0 |  |  | 8 | 30.8 |  | 10 | 38.5 |  |
|  | II | 2 | 6.1 |  | 2 | 7.4 |  |  | 0 | 0.0 |  | 2 | 7.7 |  |
|  | III | 2 | 6.1 |  | 0 | 0.0 |  |  | 2 | 7.7 |  | 0 | 0.0 |  |

*NC* neoadjuvant chemotherapy; *PDK* pyruvate dehydrogenase kinase*.*
