# Supplementary figures and images for: High hepatic expression of PDK4 improves survival upon multimodal treatment of colorectal liver metastases
Source: Br J Cancer. 2019 Feb 27;120(7):675–88. doi: 10.1038/s41416-019-0406-9 (PMC6461828; doi:10.1038/s41416-019-0406-9)

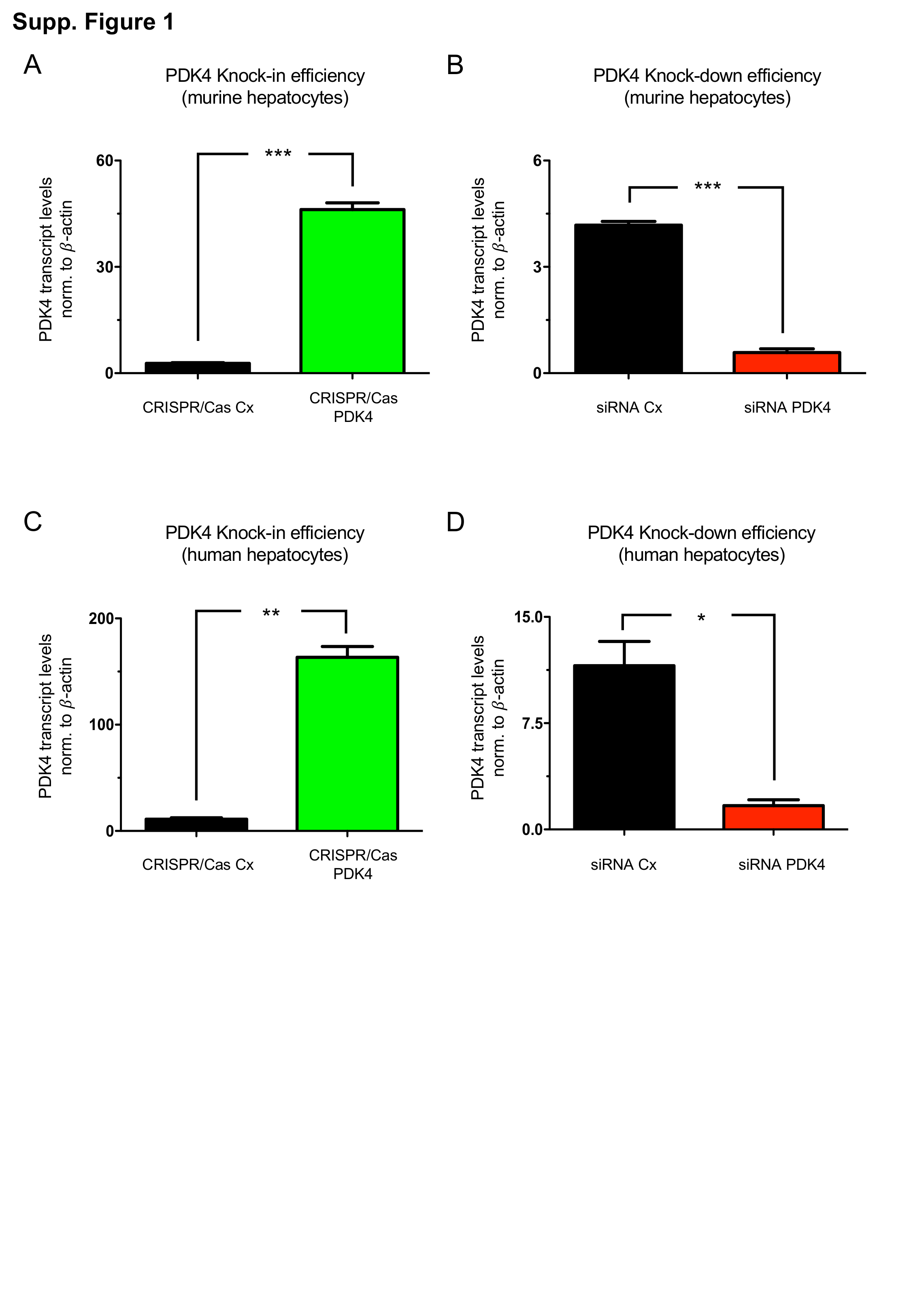

Supplement: Supplementary file 2 — Supp. Figure 1 [file 41416_2019_406_MOESM2_ESM.tif]

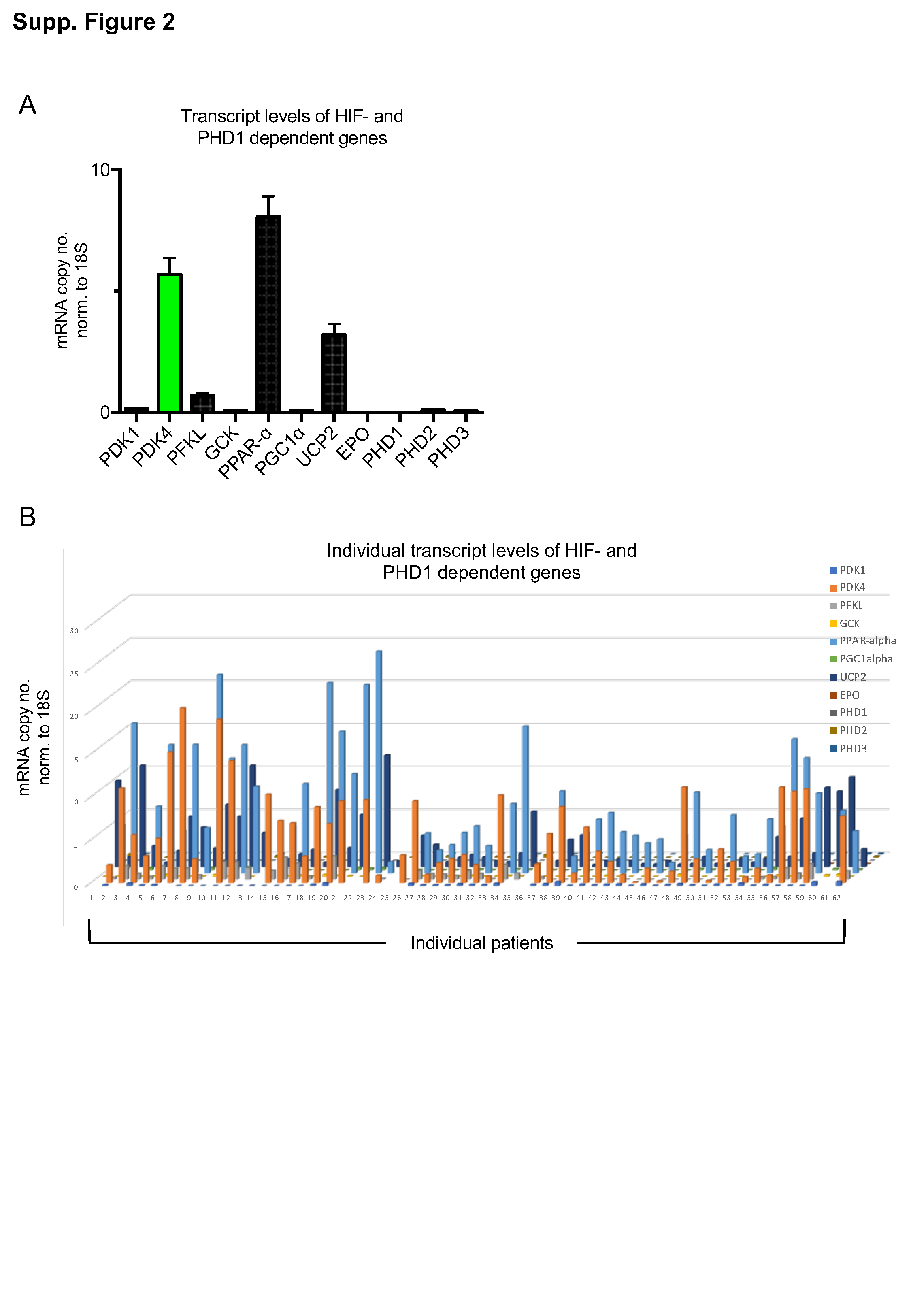

Supplement: Supplementary file 3 — Supp. Figure 2 [file 41416_2019_406_MOESM3_ESM.tif]

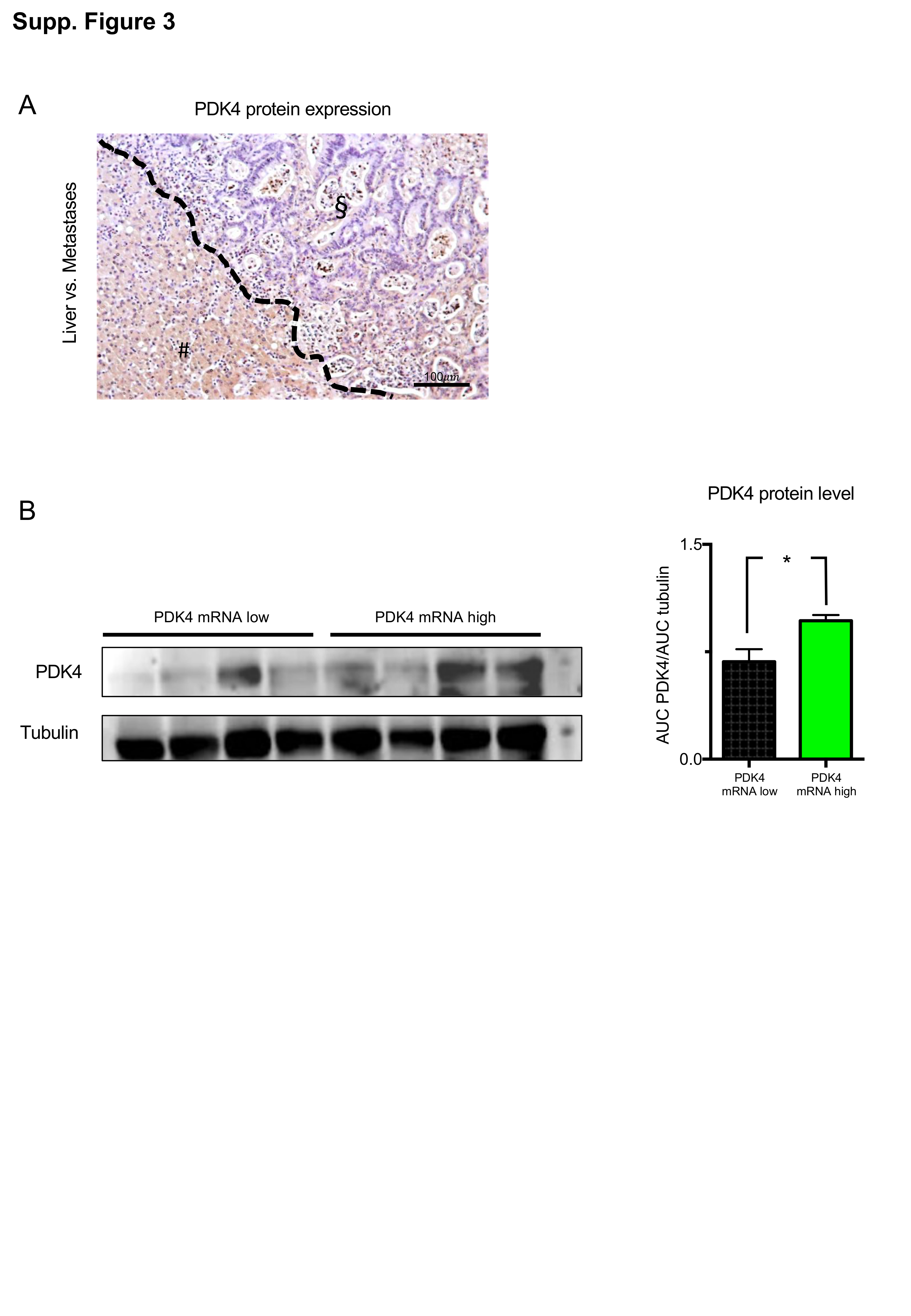

Supplement: Supplementary file 4 — Supp. Figure 3 [file 41416_2019_406_MOESM4_ESM.tif]

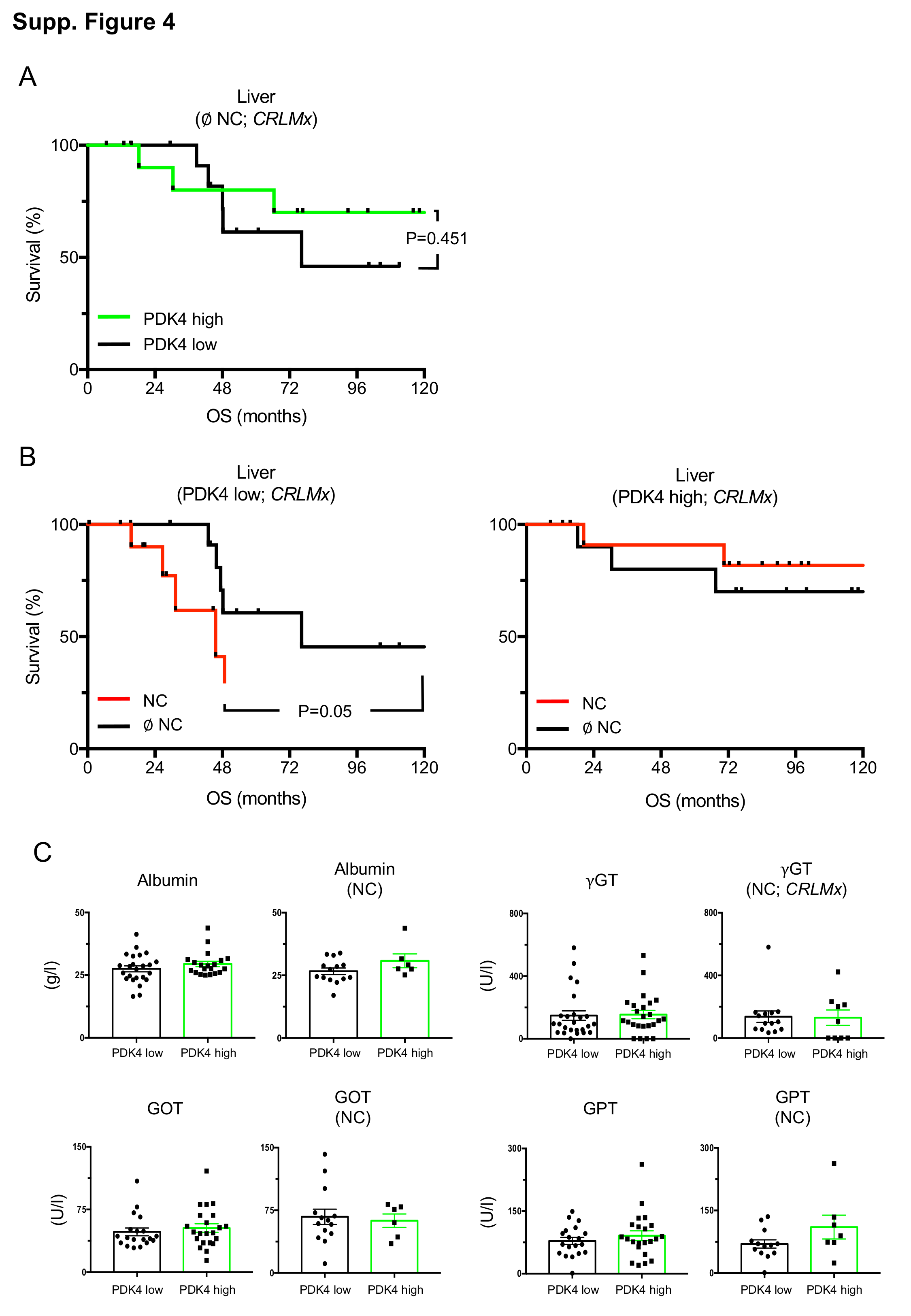

Supplement: Supplementary file 5 — Supp. Figure 4 [file 41416_2019_406_MOESM5_ESM.tif]

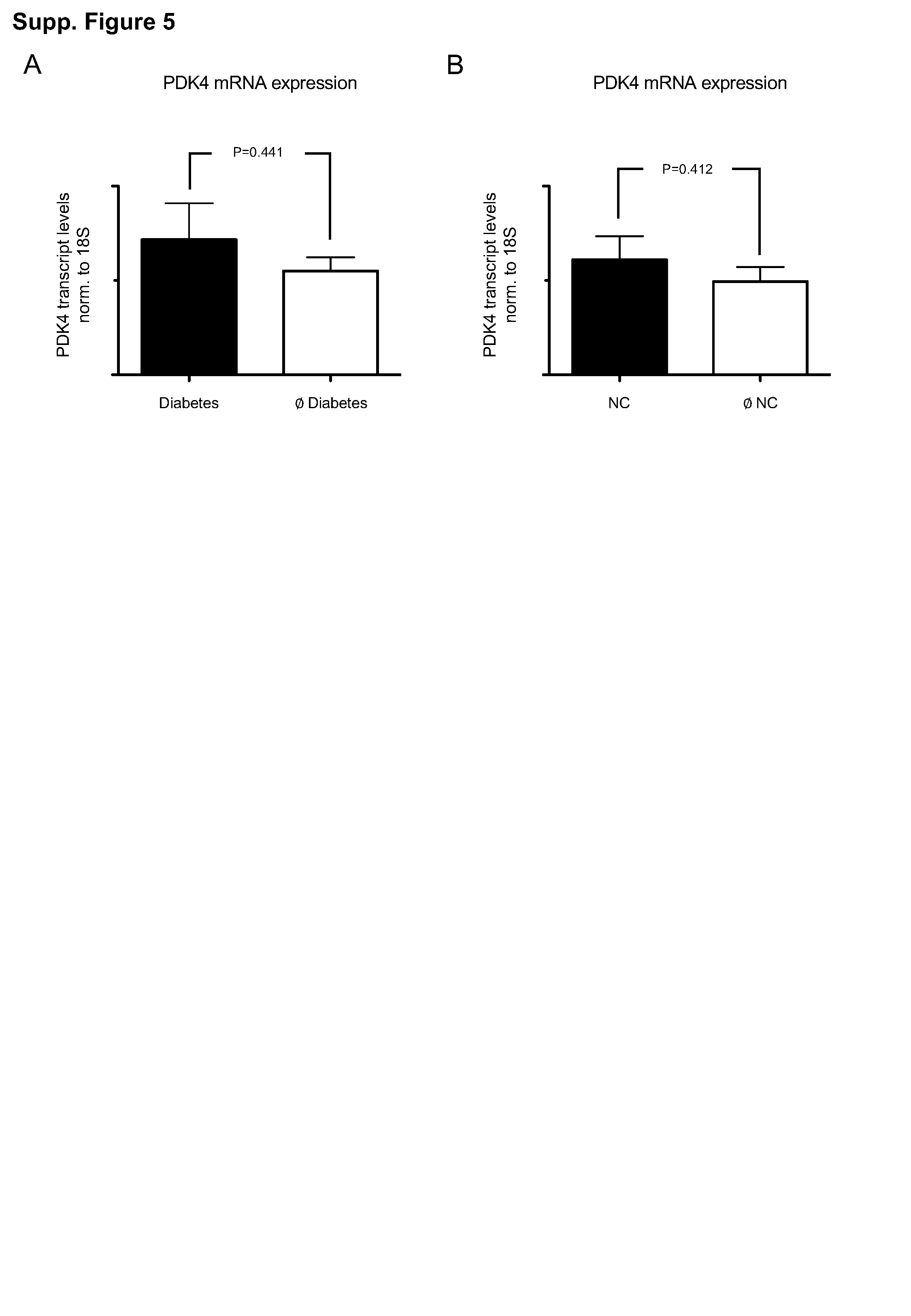

Supplement: Supplementary file 6 — Supp. Figure 5 [file 41416_2019_406_MOESM6_ESM.tif]

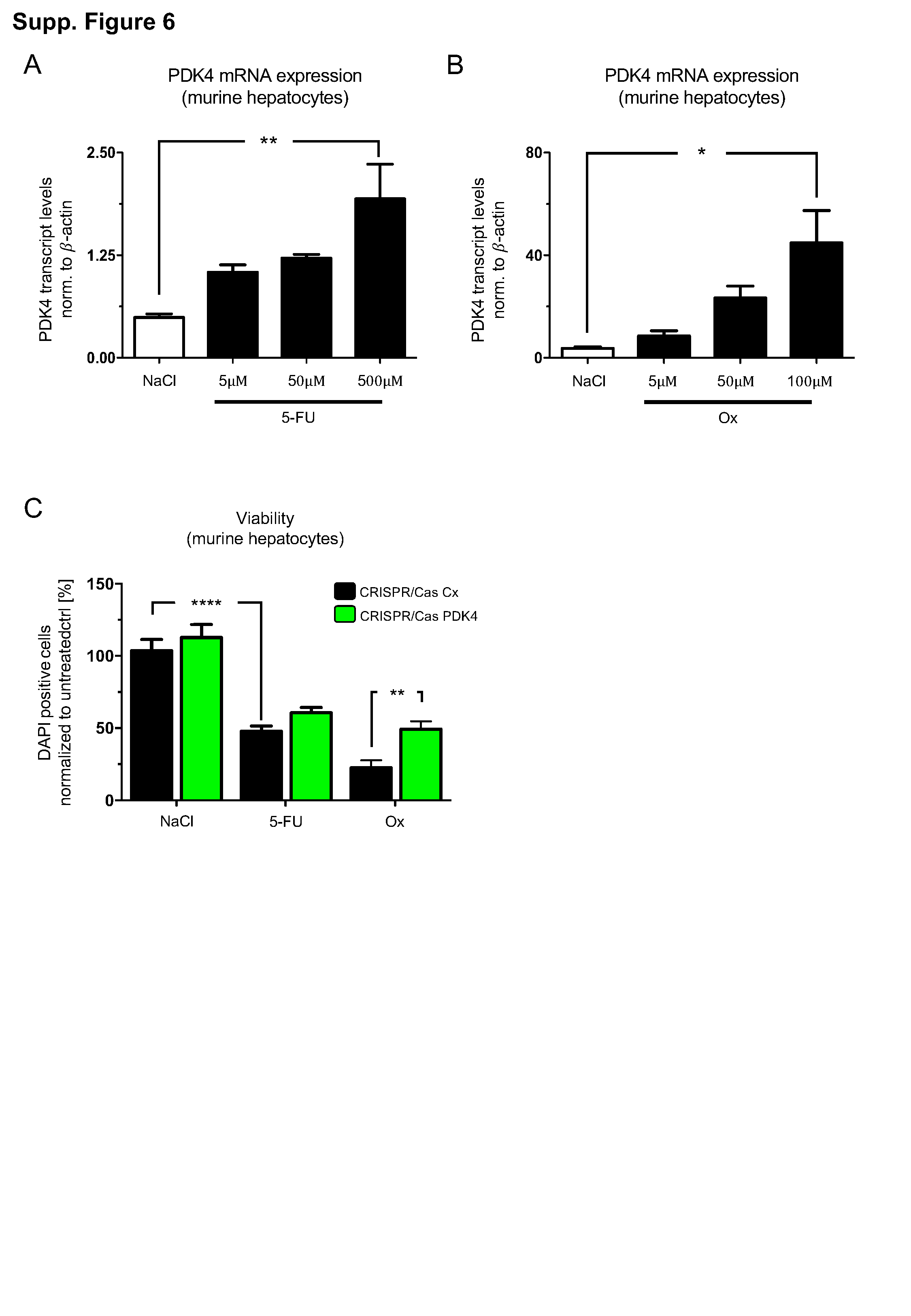

Supplement: Supplementary file 7 — Supp. Figure 6 [file 41416_2019_406_MOESM7_ESM.tif]

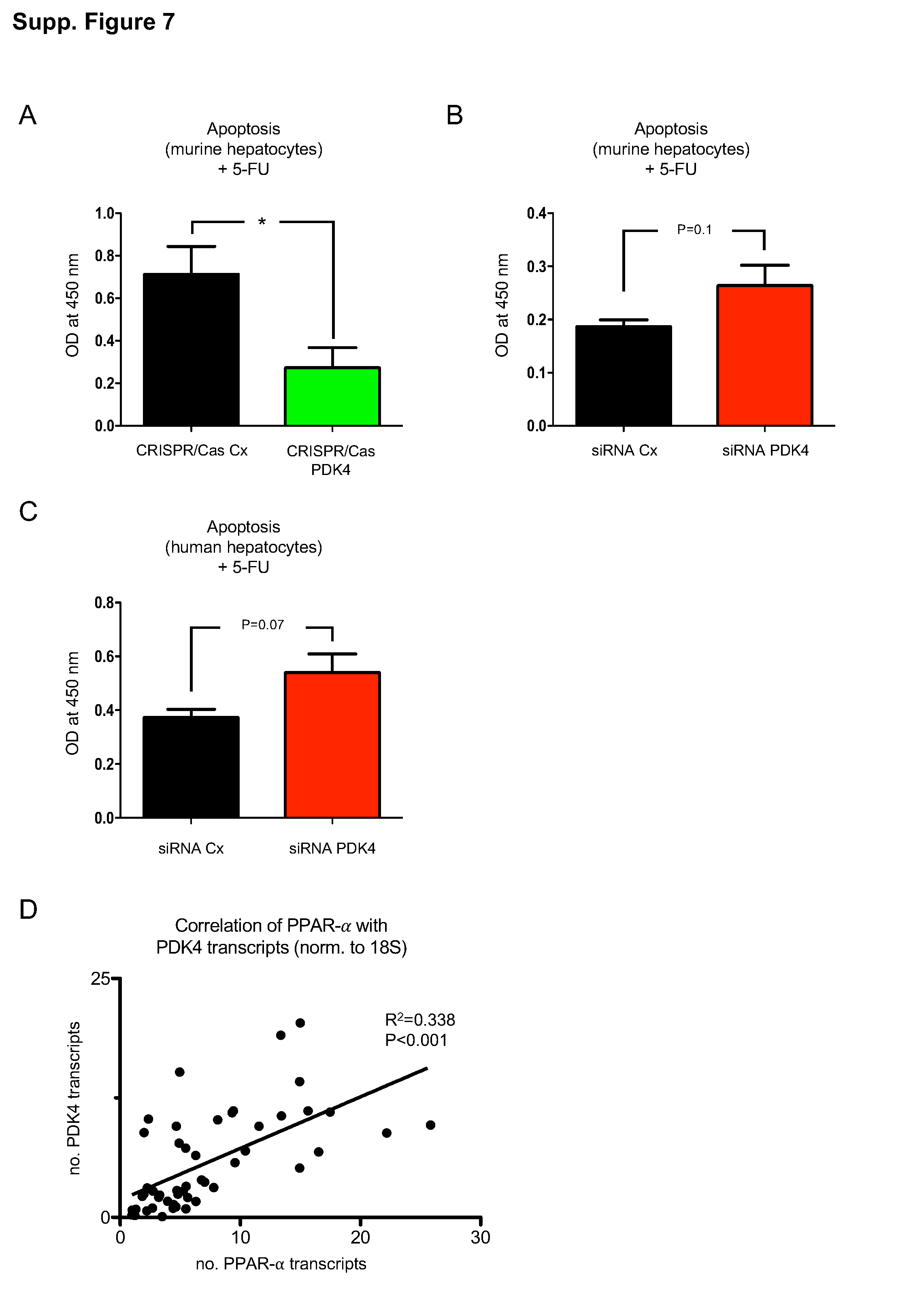

Supplement: Supplementary file 8 — Supp. Figure 7 [file 41416_2019_406_MOESM8_ESM.tif]

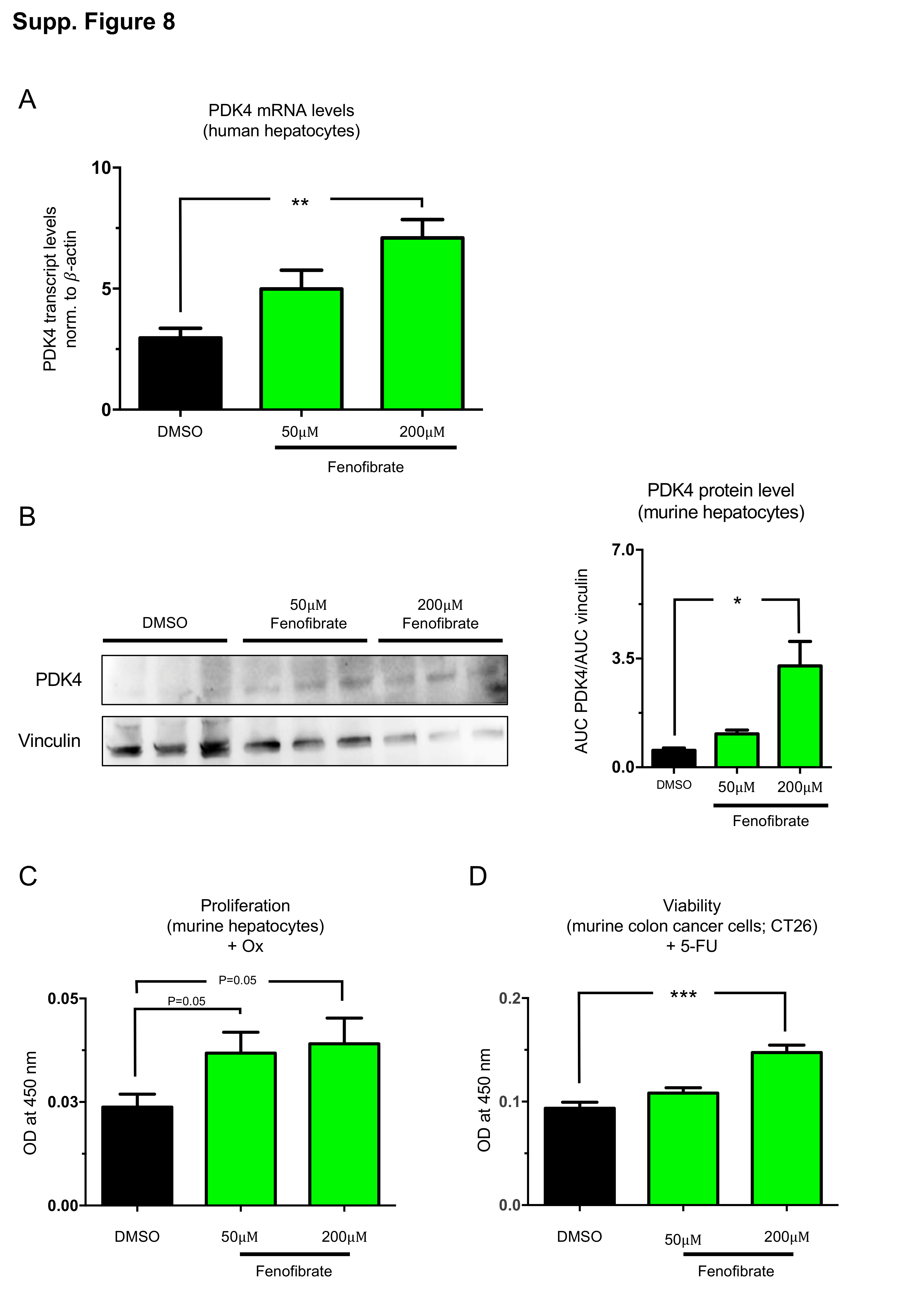

Supplement: Supplementary file 9 — Supp. Figure 8 [file 41416_2019_406_MOESM9_ESM.tif]
